# Hybridization of Curcumin Analogues with Cinnamic Acid Derivatives as Multi-Target Agents Against Alzheimer’s Disease Targets

**DOI:** 10.3390/molecules25214958

**Published:** 2020-10-26

**Authors:** Eirini Chainoglou, Argyris Siskos, Eleni Pontiki, Dimitra Hadjipavlou-Litina

**Affiliations:** Department of Pharmaceutical Chemistry, School of Pharmacy, Faculty of Health Sciences, Aristotle University of Thessaloniki, 54124 Thessaloniki, Greece; echainogg@pharm.auth.gr (E.C.); siskos@yahoo.gr (A.S.); epontiki@pharm.auth.gr (E.P.)

**Keywords:** curcumin analogues, cinnamic acids, Hybrids, Alzheimer’s disease, therapy

## Abstract

The synthesis of the new hybrids followed a hybridization with the aid of hydroxy-benzotriazole (HOBT) and 1-Ethyl-3-(3-dimethylaminopropyl)carbodiimide (EDCI.HCL) in dry DMF or thionyl chloride between curcumin analogues and cinnamic acid derivatives. IR, ^1^H-NMR, ^13^C-NMR, LC/MS ESI^+^, and elemental analysis were used for the confirmation of the structures of the novel hybrids. The lipophilicity values of compounds were calculated theoretically and experimentally via the reversed chromatography method as R_M_ values. The novel derivatives were studied through in vitro experiments for their activity as antioxidant agents and as inhibitors of lipoxygenase, cyclooxygenase-2, and acetyl-cholinesterase. All the compounds showed satisfying anti-lipid peroxidation activity of linoleic acid induced by 2,2′-azobis(2-amidinopropane) hydrochloride (AAPH). Hybrid **3e** was the most significant pleiotropic derivative, followed by **3a**. According to the predicted results, all hybrids could be easily transported, diffused, and absorbed through the blood–brain barrier (BBB). They presented good oral bioavailability and very high absorption with the exception of **3h**. No inhibition for CYP1A2, CYP2C9, CYP2C19, CYP2D6, and CYP3A4 was noticed. According to the Ames test, all the hybrids induced mutagenicity with the exception of **3d**. Efforts were conducted a) to correlate the in vitro results with the most important physicochemical properties of the structural components of the molecules and b) to clarify the correlation of actions among them to propose a possible mechanism of action. Docking studies were performed on soybean lipoxygenase (LOX) and showed hydrophobic interactions with amino acids. Docking studies on acetylcholinesterase (AChE) exhibited: (a) hydrophobic interactions with TRP281, LEU282, TYR332, PHE333, and TYR336 and (b) π-stacking interactions with TYR336.

## 1. Introduction

Alzheimer’s disease (AD) is the most common cause of dementia. According to World Alzheimer’s report 2019, over 50 million people worldwide are living with dementia, and this number is expected to be increased to more than 152 million by 2050 [1]. Multiple neurochemical pathways are involved in the pathology of AD. Several factors have been implicated in the development of AD. Thus, the rationale for multi-target therapy becomes evident when reviewing the multiple neurochemical pathways common to the disease.

AD is mainly characterized by the accumulation of amyloid-β (Aβ) plaques and neurofibrillary tangles (NFTs) of the Tau protein in the brain [2]. Chronic brain inflammation and oxidative stress also appear in AD. Acetylcholinesterase (AChE) plays a crucial role in AD patients. Recently, the role of neocortical acetylcholine (Ach) in spatial memory was defined. The discovery of the cholinergic deficit in AD underlined the role of AChE as a therapeutic target [3]. Six different classes of molecules could be useful in the medical treatment options for cholinergic deficit: (a) AChE inhibitors, increasing the synaptic levels of acetylcholine (ACh) by preventing its hydrolysis; (b) ACh precursors, such as phosphatidylcholine, enhancing the availability of choline; (c) ACh releasers, facilitating the release of ACh from presynaptic neurons; (d) M_1_ and M_3_ receptor agonists; (e) M_2_ receptor antagonists; and (f) nicotinic agonists or substances having nicotinic-like effects [4].

Mannich base derivatives with chalcone structures (enone scaffold) were found to present better AChE inhibitory activity than other compounds [5]. Thus, the design and synthesis of curcumin analogues in which the β-diketone motif is replaced by a small alicyclic amine might offer alternative AChE inhibitors.

Accumulating evidence has demonstrated that cyclooxygenase (COX) inhibitors are involved in the management of AD through their action on the down-stream effects of the insulin signaling pathway inhibiting neuro-inflammation and oxidative stress [6]. Moreover, it was previously suggested that the expression of cyclooxygenases (COXs) may affect β-amyloid peptide generation through mechanisms that involve the Prostaglandin E2 PGE2-mediated potentiation of γ-secretase activity, further supporting a role for COX-2 in the neuropathology of AD [7].

Additionally, 5-lipoxygenase (5-LOX), a pro-inflammatory enzyme widely distributed within the central nervous system, was found to be upregulated in AD [8,9]. An in vivo study also revealed that either the inhibition or knockout of 5-LOX significantly reduced γ-secretase expression and the Aβ level [10]. Hence, the 5-LOX pathway likely plays a crucial role in the development of the full pathological phenotype of AD, which includes aberrant Aβ production and deposition, as well as altered Tau phosphorylation. Based on previous studies, a specific 5-LOX inhibitor could be a significant promising molecule for the therapy of AD [11]. Since AD is a multifactorial disorder, multitarget agents have been designed and synthesized as a promising approach to target its complex etiology [12].

**Figure 1:**
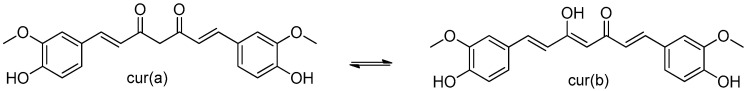
The structure of curcumin.

Curcumin (Figure 1), a natural phenolic compound extracted from the rhizome of the plant *Curcuma longa*, is of great interest to researchers due to its wide variety of bioactivities, e.g., anti-inflammatory, antioxidant, and anti-AD [13,14]. It is known that curcuminoids are classified as pan-assay interference compounds (PAINS) [15]. Many researchers have described the potential “dark side of curcumin:” poor pharmacokinetic/pharmacodynamic (PK/PD) properties, low efficacy in several disease models, and toxic effects under certain testing conditions are rendering its use significantly limited [12,16,17].

While curcumin has received the label of being pharmacodynamically fierce (hits many targets) yet pharmacokinetically feeble, these results apparently have not deterred researchers interested in the design, synthesis, and development of curcumin analogues [12,16].

In the last decade, researchers have focused on curcumin analogues with anti β-secretase or beta-site amyloid precursor protein cleaving enzyme 1 (BACE1), amyloid precursor protein APP, α- and β-secretases (Aβ fibrils aggregation), glycogen synthase kinase 3 beta (GSK-3β) (NTFs formation), metal accumulation and mitochondrial dysfunction (oxidative stress), interleukin 6 (IL-6), nuclear factor kappa (NF-kB), tumor necrosis factor-α (TNF-α), 5-LOX (neuro-inflammation), and AChE inhibition [12] properties, trying to optimize their beneficial multitarget properties against AD and improve the pharmacokinetic profile. Influential structural modifications of curcumin that improve its stability and solubility involve (i) the elimination of the hydrolysis-prone keto-enol functionality [18] (Figure 1) and (ii) the incorporation of several alternative substituents on the terminal phenyl rings or the installation of a single carbonyl group either as an acyclic agent or embedded in a small ring as 4-piperidinone. Both structural modifications avoid the extraordinarily rapid decomposition of curcumin at pH 6.5 and above in an aqueous medium and deliver improved pharmacokinetic profiles in mouse models [19].

**Figure 2:**
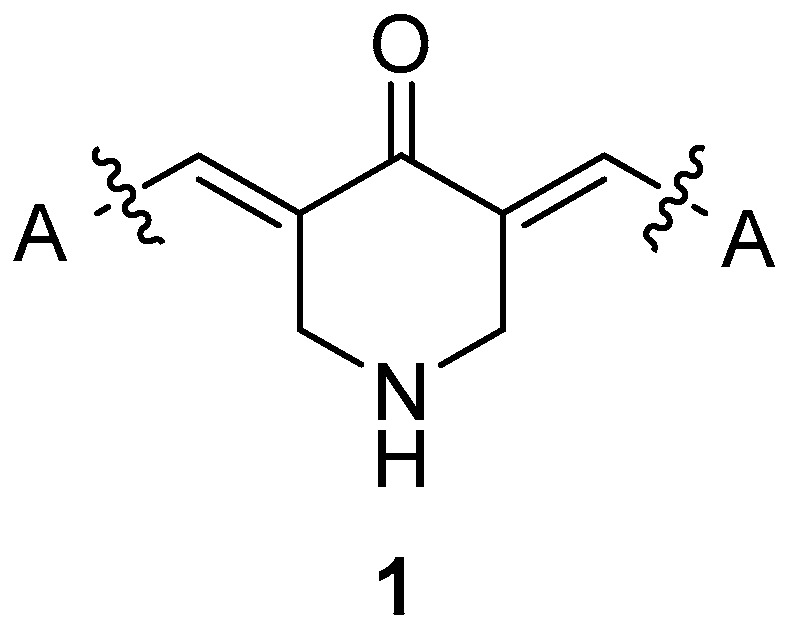
Curcumin analogue.

As a result, a series of curcumin analogues containing a mono-carbonyl moiety incorporated in a piperidone ring have been reported (Figure 2) [16]. These analogues have been found to possess high anticancer activity [20,21,22,23,24,25,26], reacting readily with thiols as Michael acceptors [27].

Ferulic, caffeic and other phenolic cinnamic acids (CAs) have been reported to present biological interest attributed to their anti-inflammatory [28,29,30], anti-oxidative [31], anti-tumor [32], anti-microbial [33], and cytoprotective activities ameliorating neuro inflammation in neurogenerative diseases [34], as well as anti-hypertensive and anti-hyperlipidemic activities minimizing the oxidation of low-density lipoprotein (LDL) [35]. Investigations on cinnamic acid derivatives developed potential new AChE inhibitors and elucidated the structure–activity relationship [36].

In the last decade, scientists have focused on multifunctional molecules instead of a single molecule hitting one target. Multi-target drugs can be beneficial for the treatment of complex and multifactorial diseases. A variety of multi-target molecules containing the cinnamic acid moiety have been reported [37,38].

Recently, for the treatment of complex diseases, hybrid drugs combining two pharmacophore moieties in one molecule have been developed. Molecular hybridization is a powerful strategy to produce multifunctional or conjugated drugs. These molecules are more medically effective than their individual components [38]. Many efforts have been made in the field of synthesis of curcumin hybrids to obtain a multifunctional drug. Characteristic curcumin hybrids are quinoline–curcumin hybrids, melatonin–curcumin hybrids, donepezil– and tacrine–curcumin hybrids, benzo[b]thiophene 1,1-dioxide (BTP)–curcumin hybrids, sulfonamide–curcumin hybrids, thalidomide–curcumin hybrids, coumarin– and isatin–curcumin hybrids [39]. Thus, we decided to design and synthesize a series of hybrids of curcumin analogues with cinnamic acid derivatives as advantageous multi-target agents.

**Figure 3:**
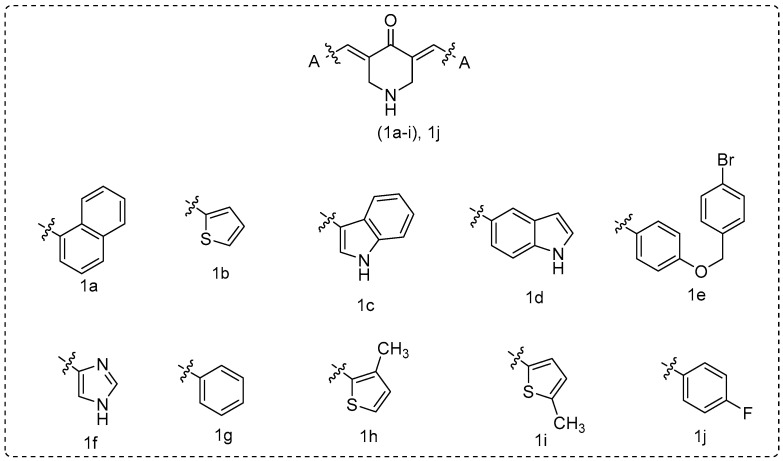
Curcumin analogues **1a**–**i** were previously reported from our research group [40] and **1j** [47,48].

During the last decade, our group has designed and synthesized curcumin analogues **1a**–**i** as anti-proliferative and anti-inflammatory agents [40,41] (Figure 3). These compounds presented LOX inhibition and anti-lipid peroxidation activity, and they were found to be implicated in NF-kB inhibition signaling pathway in L929s and A549 cell lines [41].In addition, various research groups have evaluated **1a**, **1b**, **1i**, and **1j** for antimicrobial, cytotoxic, and anti-proliferative properties [26,42,43,44,45,46,47,48] and **1a** as an inhibitor of acethylcholinesterase (AChE) and butyryl-cholinesterase (BuChE) [49,50].

**Figure 4:**
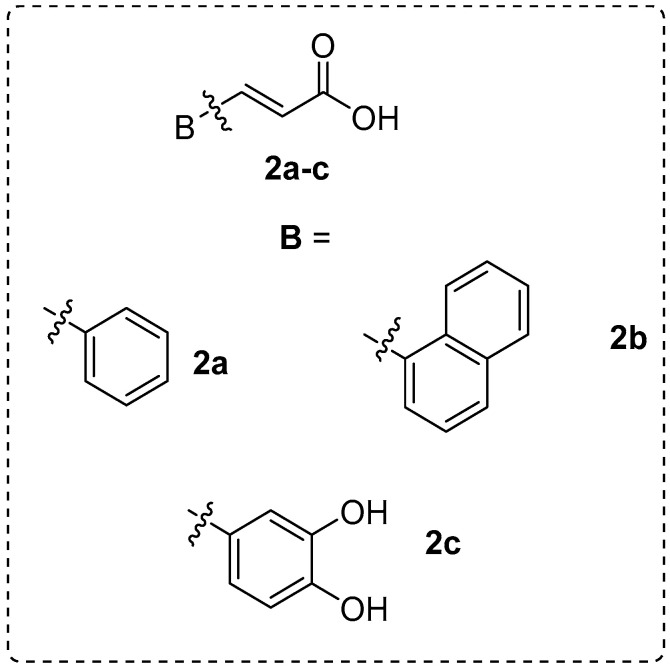
The selected cinnamic acids.

Furthermore, we have designed and synthesized several cinnamic acid derivatives as potent lipoxygenase inhibitors and antioxidant, anti-cancer, and anti-inflammatory agents (Figure 4) [37].

Earlier, Dimmock et al. [51], synthesized *N*-[4-(3-aryl-3-oxo-1-propenyl) phenylcarbonyl]-3,5-bis (phenylmethylene)-4-piperidones. All compounds were evaluated for murine toxicity, as well as for their possible penetration of the central nervous system (CNS).

The known “parent” molecule **1g** [47,52,53] and the **1a**–**i** [40] curcumin analogues (Figure 3) were synthesized and included in this study. We used modelling studies on our library structures (curcumin analogues and cinnamic acids derivatives) [28,29,30,37,38] as tools in order to design LOX, COX-2, and AChE hybrid inhibitors.

In an attempt to obtain more potent curcumin analogues to keep the biological properties and safety profile intact, we conducted biological studies on various synthetic derivatives of curcumin mainly focused on changes in the β-diketone structure and the aryl substitution scaffold. Therefore, we synthesized a number of compounds: (a) by replacing the piperidinone ring by a five-membered ring, like cyclopentanone (**1k**); (b) replacing the nitrogen in the piperidinone ring by an oxygen (**1l** and **1m**) or a sulfur (**1n**); and (c) replacing the carbonyl group of the piperidinone by a C=S group (**1o** and **1p**).

The drug-likeness of the derivatives (final hybrids and curcumin analogues) was determined from the theoretical calculation of various molecular properties. The in silico results were evaluated, and the violations of Lipinski’s rule were considered using several platforms as well as the prediction of Absorbance, Distribution, Metabolism, Elimination, Toxicology, ADMET properties and toxicity. The gathered information supported the synthesis of the hybrid compounds in an effort to define the importance and correlation of lipophilicity and the steric and electronic parameters on the biological activities and to improve them.

## 2. Results and Discussion

### 2.1. Chemistry

**Figure 5:**
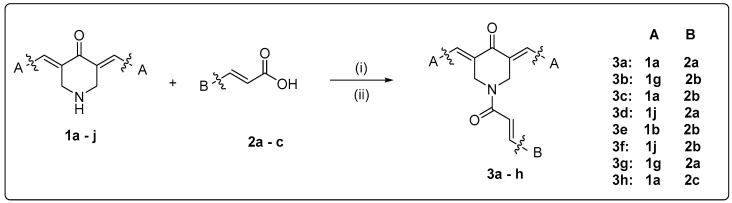
Scaffold combinations and synthetic procedure for the hybridization: (**i**) a mixture of curcumin analogue (1 eq.) with acid (1.5 eq.), HOBT (2 eq.), and EDCI.HCL (3.5 eq.) in dry DMF (3–5 mL) stirring at r.t. under nitrogen or argon gas; (**ii**) a mixture of acid (0.003 mol), thionyl chloride (0.007 mol), *N*,*N*-dimethylformamide (0.04 mL), and dry dichloroethane (40 mL) under reflux for 6 h. A curcumin analogue (0.003 mol) was added and cooled in an ice-bath for 20 min. Et3N (0.006 mol) in dichloroethane (20 mL) was added dropwise at the ice-bath temperature.

The design and synthesis of the new multitarget curcumin analogue–cinnamic hybrids were based on a combination of curcumin analogues containing a monocarbonyl moiety incorporated into a piperidone ring [40] with the amide linkage of the cinnamic acids derivatives [36,51] (Figure 5). Variations were accomplished by the choice of suitable substituted cinnamic acids of our compilation [28,29,30,37,38], as well as of suitable curcumin analogues that showed interesting biological activities in our previous [40,41] and recent work. Furthermore, new curcumin analogues, acids, and hybrids were synthesized by using our in silico results.

For the synthesis of the desired curcumin analogues **1a**–**j**, a Claisen–Schmidt condensation was accomplished [40] between the 4-piperidinehydrochloride monohydrate and the appropriate aryl aldehyde at a molar ratio of 1:2, in acetic acid by passing dry HCl gas through the mixture. All the synthesized compounds were characterized spectrophotometrically, and the results were in agreement with the previous findings [40]. The X-ray crystallography studies of previously synthesized analogues confirmed the isolation of *E*, *E* isomers [23,53]. The olefinic double bond was found to possess *E* stereo chemistry. The NH absorptions were not observed for most of the compounds in series **1**. The findings were in agreement with earlier publication [40].

The compounds **1k**–**n** and **1q** were prepared by the condensation of the appropriate ketone and arylaldehyde under basic conditions in ethanol using microwave (MW) irradiation to afford the target curcumin analogues. Compounds **1k**, **1l**, **1m**, and **1n** had been synthesized earlier under different experimental conditions [54,55,56,57]. We used a different synthetic procedure, and the structures of the known compounds were verified according to literature spectral data, elemental analysis, or mps. In all cases, our synthetic technique was simpler.

**Figure 6:**
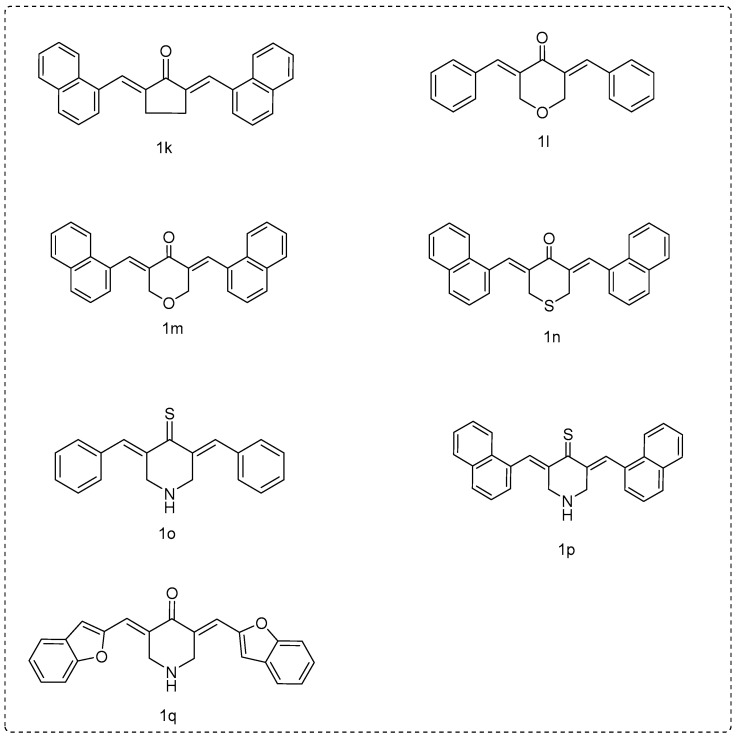
Miscellaneous curcumin analogues.

Lawesson’s reagent is a mild and convenient thionating agent for ketones, esters, and amides that allows for the preparation of thioketones, thioesters, and thioamides in good yields. Compounds **1g** and **1a** were transformed to the corresponding **1o** and **1p** using the Lawesson’s reagent [58]. Mild conditions were used. It seems that the volume of Substituent A influenced the yield of the reaction. Thus, compound **1o** resulted in a higher yield % (71%) compared to the results provided by **1p**. Spectrometric data supported the given structures (Figure 6).

The synthesis of cinnamic acids **2a**–**c** was established by the Knoevenagel–Doebner condensation of the suitable aldehyde with malonic acid in the presence of pyridine and piperidine as we have earlier reported [37].

The structural characterization of the new curcumin analogues **3a**–**h** was based on their spectral data and elemental analyses. For example, the IR spectra of compounds revealed an absorption band at 1669–1659 cm^−^ characteristic to carbonyl group of the curcumin analogue and to the amide group of the hybrid. Their ^1^H-NMR spectra revealed two signals at δ 7.67–7.96 ppm assignable to vinylic protons of benzylidenes. The survey of the ^13^C-NMR spectra of title compounds revealed that the carbonyl carbon was displayed downfield at >189 ppm and the amidic carbonyl group at >165 ppm. The LC–MS results pointed to the presence of [M + CH_3_OH]^+^, [M + CH_3_OH + Na]^+^, and [M + Na]^+^. The physicochemical properties of the novel derivatives are given in the experimental section.

### 2.2. Physicochemical Studies

#### 2.2.1. Experimental Determination of Lipophilicity as R_M_ Values

Since lipophilicity is described as a major physicochemical parameter that affects ligand–target binding interactions, solubility, ADME (absorption, distribution, bioavailability, metabolism, and elimination), and toxicological effects, we considered it important to experimentally determine this property as R_M_ values. The RPTLC (reverse phase thin layer chromatography) method, which has been characterized as a secure, rapid, and appropriate technique for expressing lipophilicity, was applied (Table 1) [37]. We tried to correlate the milog P values, the theoretically calculated lipophilicity in one equation, with the R_M_ values of all the compounds (Table 1). However this attempt was found to be unsuccessful. The perusal of the lipophilicity values of hybrids showed that **3a**, **3b**, **3e**, and **3f** are lipophilic compounds when counting the experimental/theoretical lipophilicity values. Considering the curcumin analogues, it seemed that only for **1k** and **1m** was there an agreement in both experimental/theoretical values. Hybrids **3c** and **3h** presented the lowest lipophilicity—as R_M_ values—among the hybrids (negative scale), as well as similar (−0.658/−0.657; Table 1), whereas the calculation indicated a higher lipophilicity. This disagreement could be attributed to several factors, e.g., different solvation, silanophilic interaction, H-bridges, and differences in chemical structures.

#### 2.2.2. In Silico Determination of Drug-Likeness

Drug-likeness qualitatively assesses the possibility for a molecule to become an oral drug with respect to bioavailability. Nowadays, experimental procedures for the determination of human pharmacokinetic properties have been progressively replaced by in silico procedures since the computational tools are faster, simpler, and more cost-effective [60].

Herein, we used a variety of useful in silico methods to screen the ADMET properties of our compounds for comparison reasons. We simultaneously analyzed the used curcumin analogues and the final hybrids. In this regard, we obtained and entered in the online software versions of (i) Molinspiration software version 2016.10 (www.molinspiration.com) [59], (ii) the MolSoft platform (http://molsoft.com/mprop/) [61], (iii) the CypRules platform (https://cyprules.cmdm.tw/) [62], (iv) the preADMET platform (https://preadmet.bmdrc.kr/) [63], and (v) the SwissADME platform (http://www.swissadme.ch) [64], in order to obtain the chemical structures and simplified molecular-input line-entry system (SMILES) notations of the synthesized curcumin analogues, cinnamic acids, and hybrids.

According to Lipinski’s rule, the poor absorption or permeation is related to the presence of more than 5 H-bond donors and 10 H-bond acceptors. Furthermore, values of the molecular weight (MW) > 500 and calculated log *P* value > 5 lead to poor absorption/permeability. We noticed that all hybrids (**3a**–**h**) presented high lipophilicity values and MWs (Table 2).

We calculated the molecular properties predictors via the MolSoft platform (Table SI, Supplementary Material). The percentage of absorption (%ABS) was calculated by using %ABS = 109 − (0.345 × topological polar surface area (TPSA)) [65] and is referred to as the degree of absorption. With the exception of hybrid **3h** and the analogue **1f**, all the other hybrids presented a very high absorption—higher than curcumin. The log S values were found to be very low compared to the corresponding curcumin (Table SI, Supplementary Material).

All the curcumin analogues also presented high absorption % values. The bioavailability of bioactive molecules is characterized by their TPSA. This descriptor is highly correlated with the hydrogen bonding properties of a molecule. The TPSA values of the hybrids were observed in the range of 34.38–77.84 Å^2^, whereas curcumin analogues **1a**–**q** showed TPSA values 11.17–44.60 Å^2^ (**1f** with 68.44 was the only exception). These values were lower the limit of 160 Å^2^ underlying a good oral bioavailability. The upper limit for the TPSA for a molecule to penetrate the brain is around 90 Å^2^. Thus, all these molecules are able to penetrate the brain (Table 2).

LogBB is important in silico parameter to identify CNS-active agents. Since these hybrids were designed as agents to target Alzheimer’s disease, we theoretically calculated logBB values using the log*P* and TPSA values and the following equation.

logBB = 0.155clog*P* − 0.01TPSA + 0.164 (1)

Compounds with logBB values higher than 0.3 are considered to have a high absorption through the blood–brain barrier (BBB), and logBB values between 0.3 and −0.1 and lower are not considered to be absorbed in general through the BBB [66]. It seems that all hybrids could be easily transported, diffused, and absorbed through the BBB. The BBB biological scores ranged from 3.27 to 5.74 (**3h** presented a value of 2.45), whereas curcumin showed a score of 2.83. The prediction of CYP metabolism and excretion of the hybrids using the CypRules showed no inhibition for the CYP1A2, CYP2C9, CYP2C19, CYP2D6, and CYP3A4. Similarities were observed among the results using the SwissADME platform (Table 3).

The toxicity assessment of the synthesized compounds was calculated by the preADMET platform. PreADMET predicts toxicity to TA98, TA100, and TA1535 (strains of the bacterium *Salmonella typhimurium* that carry mutations in genes involved in histidine synthesis) that are often used in the Ames test. The actual value of the prediction result was “positive” (change of population) or “negative” (no change of population). With the exception of **3d**, all the other hybrids induced mutagenicity according to the Ames test. Using PreADMET, we predicted the carcinogenicity for the synthesized hybrids. The results from this model, which were built from the data of NTP (National Toxicology Program) and US FDA, were the results of the in vivo carcinogenicity tests of mice and rats for two years. “Negative” suggests clear evidence of carcinogenic activity, and “Positive” underlines the absence—no evidence of carcinogenic activity. Only **3d** gave a positive sign in mice, whereas a negative one was observed for rats. However, we could see that although **3a**, **3b**, **3c**, and **3g** hybrids were predicted with positive sign to the rat assay, the calculation pointed to negativity against mice (Table 4). In the future, we will try to perform in vivo experiments in order to define the carcinogenicity of the most potent compound.

The SwissADME, Ghose (Amgen), Veber (GSK), Egan (Pharmacia), and Muegge (Bayer) methods give multiple estimations that allow for consensus views or the selection of methods best fitting the end-user’s specific needs in terms of chemical space. In Table 5, we have accordingly provided multiple estimations of drug-likeness.

### 2.3. Biological Evaluation

In this research, the curcumin analogues and the novel hybrids were evaluated in vitro as pleitropic agents, e.g., inhibitors of (i) soybean lipoxygenase, bovine COX-2, eel AChE, and (ii) lipid peroxidation.

Cell metabolism is the source for the continuously production of reactive oxygen species (ROS). Some are characterized as highly toxic, and various cellular enzymatic and non-enzymatic mechanisms offer a rapid detoxification. Their extreme reactivity and the tendency to induce chain reactions lead to pathological processes. Earlier studies have shown a significant enhancement of lipid peroxidation in the brain of Alzheimer’s patients. Thus, antioxidants inhibiting brain lipid peroxidation could aid their treatment. We found it interesting to determine their anti-lipid peroxidation activities in comparison to a well-known antioxidant, i.e., Trolox (Table 3).

We used the water-soluble 2,2′-azobis(2-amidinopropane) hydrochloride (AAPH) to generate in vitro peroxyl radicals through spontaneous thermal decomposition. The derived experimental conditions resembled cellular lipid peroxidation due to the activity of the undertaken radicals. The **1k**, **1m**, **1n**, and **1p** compounds (100%) were the most active inhibitors among the curcumin analogues, whereas **1j**, **1l**, **1o**, and **1q** exhibited moderate or very low activity at 100 µM concentration (8–42%). It seemed that lipophilicity was the main influence on anti-lipid peroxidation. A higher lipophilicity leads to higher activity. Thus, **1l**, which has a lipophilicity value of 3.91, and **1o**, which has a lipophilicity value of 4.62, presented lower activity (42% and 8%, respectively) compared to **1m** with a value of 5.87 (100%), **1k** with 6.42 (100%), and **1p** with 6.58 (100%). The nature of the six-membered heteroatom ring did not seem to influence the antioxidant ability, e.g., as in curcumin derivatives **1m** and **1p**. Both presented a 100% anti-lipid peroxidation. The cinnamic acids (**2a**–**c**) seemed to be equipotent. Overall, the hybrids presented a high anti-lipid peroxidation activity (84–93%; Table 3), with the exception of **3g** (53%).

Chu and Pratico [67] defined the role of 5-lipoxygenase as an endogenous modulator of amyloid beta formation. It seems that in AD brains, 5-LOX protein levels that are higher than those presented in healthy controls are accumulated, [67]. The activation of brain lipoxygenases is an early event in the pathogenesis of Alzheimer’s disease [68]. Novel research has underlined a new role for 5-LOX in regulating endogenous Tau metabolism in the central nervous system, thus supporting the theory that the inhibition of 5-LOX could be beneficial for Tau neuropathology [69].

Due to a lack of sufficiently-purified human LOX enzymes, most research has been done on soybean LOX [70,71]. Experimental results have shown a sufficient qualitative correlation between inhibition values of the two LOX enzymes [72,73,74]. In our assays, we used isozyme LOX-1, which uses free fatty acids as substrates [71] and presents maximal activity at pH 9.0, producing a conjugated diene absorbing at 234 nm by the conversion of linoleic acid into to 13-hydroperoxylinoleic acid [75].

A perusal of the %/IC_50_ (Inhibitory concentration) LOX inhibition values (Table 3) showed that curcumin analogues **1o** and **1k** were the most potent, followed by **1p**. Considering the structural characteristics, we could see that **1k** > **1p** > **1b** > **1m** > **1n**. It seemed that within the derivatives of the naphthyl substituted curcumin analogues, the potency was related with the nature of the five- or six-membered ring. The cyclopentanone led to a potent LOX inhibitor. In continuation, among the six-membered substituted analogues, the carbonyl group in compound **1p** had been replaced by a C=S group. Compound **1b** presented an IC_50_ value very close to the previous analogue. Replacement by a tetrahydro-4*H*-pyranone (**1m**) or by a tetrahydro-4*H*-thiopyranone ring (**1n**) diminished the activity.

Analogues **1l** and **1o** presented very low activities (<10%). The higher lipophilicity of **1p** (6.58) compared to **1o** (4.62) led to higher inhibitory activity. For the sake of comparison, the previously published results for **1a**–**g** compounds [16] were considered. It seemed that the presence of heterocyclic rings as substituents in Substituent A did not positively affect the activity (**1a**–**g** and **1q**). Among the cinnamic acids **2a**–**c**, the most active LOX inhibitor was **2c**, followed by **2b** and **2a** [37]. In our experiments, the hybrids exhibited IC_50_ values ranged from 34 to 82 µM. Among the tested compounds, the most interesting representatives were **3h** and **3c**. It is known that LOXs contain a “non-heme” iron per molecule in the enzyme-active site. As a consequence, LOX inhibitors (a) can reduce the iron species in the active site to the catalytically inactive ferrous form or (b) can act as ligands for Fe^3^. Hybrid **3h** contained the scaffold of caffeic acid and might act as a ligand for Fe^3^ through the two vicinal hydroxyl moieties. The literature gives evidence for a positive role of lipophilicity for LOX inhibitors [76]. However, in this dataset, lipophilicity did not seem to be correlated with LOX inhibition (theoretical lipophilicity values milog P **3h** < **3c**; experimental R_M_ values were found to be similar **3c** = **3h**).

We tested, in vitro, the inhibitory activity of the compounds on acetylcholinesterase activity using acetyl-thiocholine as a substrate [77]. The ability of these compounds to act as acetylcholinesterase inhibitors can help AD patients and may contribute to the mechanisms of action of reported structurally related curcumin analogues and/or cinnamic acids derivatives [12]. Among the tested hybrids, the most active representatives were **3e** > **3a** > **3g** (Table 3). In all cases, the moiety of cinnamic acid corresponded to the substituted naphthyl. The thienyl curcumin analogue led to the more potent hybrid (**3e**). Hybrids **3b**, **3c**, **3d**, **3f**, and **3h** showed a very low activity (14–34%). The % inhibition values for the curcumin analogues given in Table 3 were of low importance (2–32%). Cinnamic acids also did not exhibit anti-cholinesterase activity.

A perusal of logP (Table 1) and IC_50_ or %AChE values in Table 6 reveal that the role of lipophilicity on the inhibition of AChE was also not well-defined in this series of compounds.

The pathogenesis of Alzheimer’s disease (AD) is widely associated with COX-2. We studied our compounds in vitro. No activity was observed by the tested curcumin analogues. Among the cinnamic acids, caffeic acid presented anti-COX-2 activity. Within the hybrids, only **3e** showed 19% inhibition (Table 6).

Considering our results, the above LOX and COX-2 inhibitors are thought to protect against the inflammatory reactions that are known to be present in the neurons of patients with AD. A large amount of positive data have been reported on the use of NSAIDs in the treatment of AD in the literature [78]. The recorded antioxidant effects and AChE inhibition can improve existing cognitive functions and prevent current symptoms from worsening. A potential anti-AD treatment may be developed based on the multi-functional activity of the tested compounds.

### 2.4. Computational Studies—Docking Simulation Soybean Lipoxygenase

#### 2.4.1. Putative Binding Modes of Curcumin Analogues **1k** (Blue), **1l** (Purple), **1m** (Green), and **1o** (Pink) in Soybean LOX.

**Figure 7:**
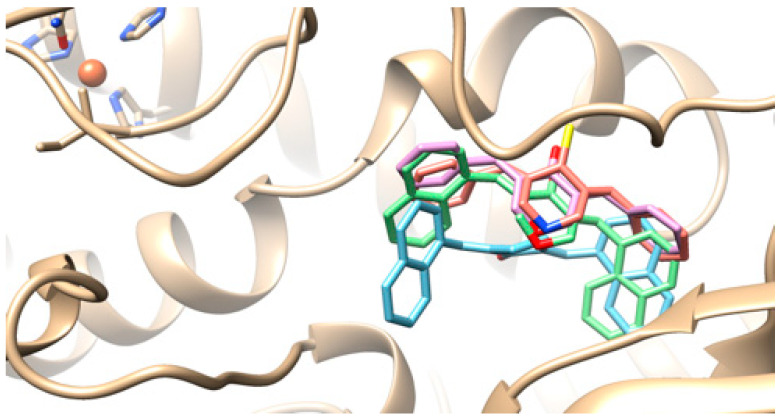
Putative binding modes of compounds **1k** (blue), **1l** (purple), **1m** (green), and **1o** (pink).

It seems that these curcumin analogues interacted with Soybean Lipoxygenase SLOX in the same way with an allosteric binding mode. There was a superposition between the cyclopenta-1-one, tetrahydro-4H-pyran-4-one, and piperidine-4-thione central rings and their 2-napthyl and benzyl substituents (Figure 7). Docking scores were: **1k**: −11.1; **1l:** −9.4; **1m**: 12; and **1o**: −8.9 kcal/mol.

#### 2.4.2. Molecular Modeling of the Synthesized Derivatives in Soybean LOX

**Figure 8:**
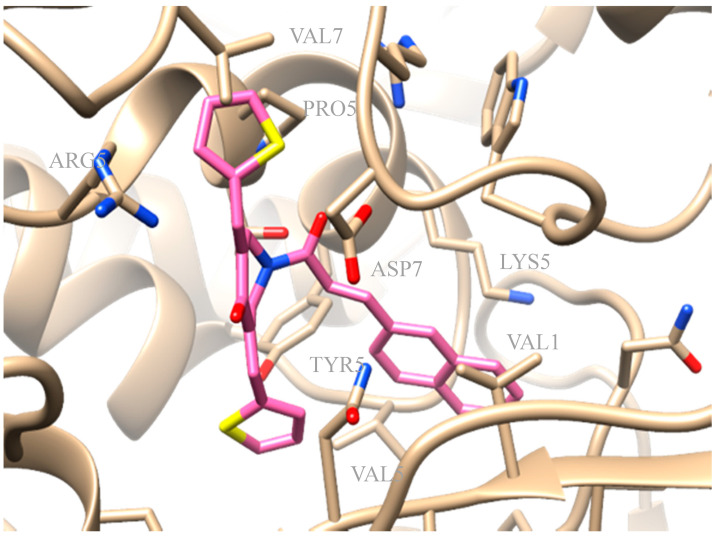
Preferred docking pose of **3e** (depicted in pink) bound to soybean lipoxygenase (LOX-1).

The synthesized derivatives were subjected to in silico studies. The favored docking position of compound **3e** is shown in Figure 8. Compound **3e**, presenting IC_50_ = 52µM of LOX inhibition in vitro, had an AutoDock Vina score of −10.1 kcal/mol binding to soybean LOX (PDB code: 3PZW). Docking scores were based on algorithms and scoring function calculations, so a one-to-one correlation is difficult to be reached, whereas biological results from the in vitro inhibition of soybean lipoxygenase represent experimental values. Docking results describe the ligand binding mode to the protein and their interactions. It seems that the novel compounds interacted with the SLOX through allosteric interactions. Compound **3e** seemed to develop hydrophobic interactions with amino acids VAL126, VAL520, TYR525, PRO530, ARG533, VAL762, and ASP768 and π-cation interactions with LYS526. From the literature, it is known that LOX inhibitors act as antioxidants by scavenging free radicals [79], and the oxidation of the enzyme occurs via a carbon-centered radical on a lipid chain. Based on this fact, it can be concluded that compound **3e** probably blocks the substrate to the binding site since it extends into the hydrophobic domain, thus preventing oxidation.

#### 2.4.3. Molecular Docking Studies of the Novel Derivatives on AChE

**Figure 9:**
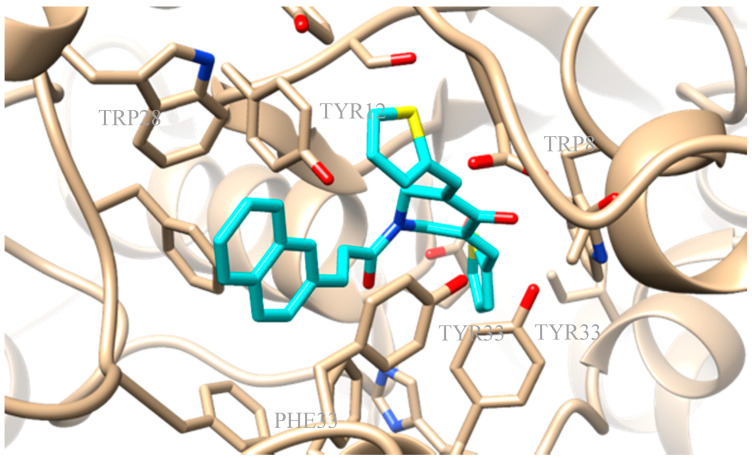
Preferred docking pose of **3e** (depicted in cyan) bound to optimized complex 4EY7.

AChE from *Electrophorus electricus* was selected for the biological assay due to its homology to human AChE. Running an alignment through UniProt with the Clustal Omega program of the AChE from *E. electricus* showed a 60.4% identity, a 74.2% positive, a 633 query length, and a 614 match length with AChE from *Homo sapiens* [80]. Molecular docking was performed for all the novel derivatives to the four optimized complexes derived from the alignment of the I-TASSER-generated 3D-model of AChE with experimental 3D-protein structures from the PDB (4BDT.A, 4EY5.A, 4EY6.A, and 4EY7.A) and subsequent minimization. Compound **3e** presented a docking score of −12.6kcal/mol to the optimized complex 4EY6. Compound **3e** exhibited a) hydrophobic interactions with amino acids TRP85, TYR123, TYR332, PHE333, and TYR336 and b) π-stacking interactions with TRP85, TRP281, and TYR336 (Figure 9).

## 3. Experimental Section

### 3.1. Materials and Instruments

All chemicals, solvents, and chemical and biochemical reagents were of analytical grade and purchased from commercial sources (Merck, Merck KGaA, Darmstadt, Germany, Fluka Sigma-Aldrich Laborchemikalien GmbH, Hannover, Germany, Alfa Aesar, Karlsruhe, Germany and Sigma, St. Louis, MO, USA). Soybean lipoxygenase, sodium linoleate, and 2,2-azobis-(2-amidinopropane) dihydrochloride (AAPH) were obtained from Sigma Chemical, Co. (St. Louis, MO, USA). Acetylcholinesterase from *Electrophorus electricus* (electric eel), Type VI-S, lyophilized powder, 200-1,000 units/mg protein, was purchased from Sigma-Aldrich, Cayman. All starting materials were used without further purification. Melting points were determined on a MEL-Temp II (Lab. Devices, Holliston, MA, USA). For the in vitro tests, UV–VIS spectra were obtained on a Perkin-Elmer 554 double-beam spectrophotometer (Perkin-Elmer Corporation Ltd., Lane Beaconsfield, Bucks, UK). Infrared spectra (KBr pellets) were recorded with Perkin-Elmer 597 spectrophotometer (Perkin-Elmer Corporation Ltd., Lane Beaconsfield, Bucks, UK). The ^1^H Nucleic Magnetic Resonance (H-NMR) spectra were recorded at 500 MHz in CDCl3 on an Agilent 500/54 (DD2) using tetramethylsilane as an internal standard unless otherwise stated. Agilent 500/54 (DD2) ^13^C-NMR spectra were obtained at 125 MHz in CDCl_3_ solutions with tetramethylsilane as internal reference unless otherwise stated. Additionally, the ^1^H Nucleic Magnetic Resonance (H-NMR) spectra were recorded at 300 MHz on a Bruker AM-300 spectrometer (Bruker Analytische Messtechnik GmbH, Rheinstetten, Germany) in CDCl_3_ or DMSO using tetramethylsilane as an internal standard. Chemical shifts are expressed in ppm and coupling constants J in Hz. Mass spectra were determined on an LC–MS 2010 EV Shimadzu (Shimadzu, Kiyoto, Japan) using MeOH as the solvent. Elemental analyses for C, H, and N gave values acceptably close to the theoretical values (±0.4%) in a Perkin-Elmer 240B CHN analyzer (Perkin-Elmer Corporation Ltd., Lane Beaconsfield, Bucks, UK). Reactions were monitored by thin layer chromatography on 5554 F254 silica gel/TLC cards (Merck and Fluka Chemie GmbH Buchs, Steinheim, Switzerland). For preparative thin layer chromatography (prep TLC), 2 mm silica gel 60 F254 plates—Merck KGaA ICH078057—were used. For the experimental determination of the lipophilicity using reverse phase thin layer chromatography (RP-TLC), TLC-Silica gel 60 F254 DC Kieselgel, Merck (Merck, Merck KGaA, Darmstadt, Germany) (20 × 20 cm) plates were used.

### 3.2. Chemistry General Procedures

#### 3.2.1. Synthesis of Curcumin Analogues **1a**–**q**

Two different methods were used for the synthesis of the compounds.

##### General Method A

For the synthesis of curcumin analogues **1a**–**j [16]**, the compounds were prepared by a Claisen–Schmidt condensation between the 4-piperidinone hydrochloride monohydrate and the appropriate aryl aldehyde at a molar ratio of 1:2 in acetic acid. Dry hydrochloride gas was passed through the mixture for at least 1 h, according to our previous published methodology [40,53].

*(3E,5E)-3,5-bis(naphthalen-1-ylmethylene)piperidin-4-one* (**1a**) [40].
*(3E,5E)-3,5-bis(thiophen-2-ylmethylene)piperidin-4-one* (**1b**) [40].
*(3E,5E)-3,5-bis((1H-indol-3-yl)methylene)piperidin-4-one* (**1c**) [40].
*(3E,5E)-3,5-bis((1H-indol-5-yl)methylene)piperidin-4-one* (**1d**) [40].
*(3E,5E)-3,5-bis(4-((4-bromophenoxy)methyl)benzylidene)piperidin-4-one* (**1e**) [40].
*(3E,5E)-3,5-bis((1H-imidazol-2-yl)methylene)piperidin-4-one* (**1f**) [40].
*(3E,5E)-3,5-dibenzylidenepiperidin-4-one* (**1g**) [40].
*(3E,5E)-3,5-bis((3-methylthiophen-2-yl)methylene)piperidin-4-one* (**1h**) [40].
*(3E,5E)-3,5-bis((5-methylthiophen-2-yl)methylene)piperidin-4-one* (**1i**) [40].
*(3E,5E)-3,5-bis(4-fluorobenzylidene)piperidin-4-one* (**1j**) [40,48].

##### General Method B—Synthesis of Curcumin Analogues **1k**–**n** Using Microwave (MW) Irradiation

An aldol condensation between the appropriate alicyclic ketone (cyclopentanone, tetrahydro-4*H*-pyran-4-one,tetrahydro-4*H*-thiopyran-4-one, or 4-piperidinone hydrochloride monohydrate) and the appropriate aryl aldehyde was performed at a molar ratio of 1:2, respectively, in 3 mL of ethanol and 200 µL of NaOH (40% *w*/*v*) in a 10 mL reaction vial containing a stirring bar under MW irradiation in a CEM-Discover mono mode microwave device operating at a frequency of 2.45 GHz (using 100 watts as the maximum power at 40 °C for 60 min and 100 psi). The vial was sealed with a Teflon septum and placed into the microwave cavity. At the end of the reaction, the mixture was rapidly cooled with gas jet cooling to room temperature.

The progress of the reaction was monitored using TLC. The separated solid was filtered, washed with water, and recrystallized from 95% aqueous ethanol.

##### General Method C—Synthesis of Curcumin Analogues **1o**–**p**

Compounds **1a** and **1g** were transformed to the corresponding **1o** and **1p** using Lawesson’s reagent; 0.56 mmoles of **1a** or **1g** in dry toluene (10 mL) were added to Lawesson’s reagent (1.13 mmoles) dissolved in 50 mL of dry toluene at r.t. The mixture was stirred at r.t. until the reaction was completed, for at least 24 h (TLC monitoring). The solvent was evaporated, and then the residue was washed with water and extracted with acetic ethyl ester and dried over Na_2_SO_4_. The solvent was evaporated. Recrystallisation followed.

*(2E,5E)-2,5-bis(naphthalen-1-ylmethylene)cyclopentanone* (**1k**): According to general method B, cyclopentanone and naphthyl-aldehyde-1 were used at a molar ratio of 1:2 in 3 mL of ethanol and 200 µL of NaOH (40% *w*/*v*) under MW, as described above. Yield: 34%; R_f_ = (hexane:acetone, 1:1): 0.73; m.p.: 101–105 °C; IR (Nujol, cm^−1^): 1670; ^1^H NMR (500 MHz, CDCI_3_) δ (ppm): 8.41 (s, 1H), 8.26 (d, *J* = 8.1 Hz, 1H), 7.89 (d, *J* = 4.9 Hz, 4H), 7.68 (d, *J* = 6.7 Hz, 1H), 7.50–7.58 (m, 9H), 3.05–3.23 (br, 4H); ^13^C NMR (125 MHz, CDCI_3_) δ (ppm): 195.7 (C=O), 143.6, 143.0, 139.8 133.5, 133.6, 132.4, 132.2, 132.0, 130.5, 129.7, 129.5, 129.4, 128.6, 128.3, 127.1, 127.0, 126.7, 126.5, 126.4, 126.2, 126.1, 125.5, 125.0, 124.0, 122.9, 27.0. Anal. C, H. Elemental Analysis: Expected % (C_27_H_20_O): C, 89.97; H, 5.59; Found %: C, 89.82; H, 5.69; *m*/*z* (ESI positive) 415.5 [M + CH_3_OH + Na]^+^.

*(3E,5E)-3,5-dibenzylidenedihydro-2H-pyran-4(3H)-one* (**1l**): According to general method B, tetrahydro-*4H*-pyran-4-one and benzaldehyde were used at a molar ratio of 1:2, in 3 mL of ethanol and 200 µL of NaOH (40% *w*/*v*) under MW, as described above. The compound has been previously synthesized [50,51,52]. A different method has been reported. Yield: 68.5%; R_f_ = (hexane:acetone, 1:1): 0.76; m.p.: 170–172 °C; IR (Nujol, cm^−1^): 1675; ^1^H NMR (500 MHz, CDCI_3_) δ (ppm): 7.85 (s, 1H) (-C=C), 7.38–7.45 (m, 10H), 7.33 (d, *J* = 7.1 Hz, 1H), 4.04–4.19 (s, 4H) (C-O-C). ^13^C NMR (125 MHz, CDCI_3_) δ (ppm): 185.5 (C=O), 136.4, 134.7, 133.1, 130.4, 129.4, 128.7, 68.6 (C-O-C); Anal. C, H. Elemental Analysis: Expected %(C_19_H_16_O_2_): C, 82.58; H, 5.84; Found %: C, 82.38; H, 5.94; *m*/*z* (ESI positive) 308.3 [M + CH_3_OH]^+^. The spectral data were in agreement with the literature data.

*(3E,5E)-3,5-bis(naphthalen-1-ylmethylene)dihydro-2H-pyran-4(3H)-one* (**1m**): According to general method B, tetrahydro-*4H*-pyran-4-one and naphthyl-aldehyde-1 were used at a molar ratio of 1:2 in 3 mL of ethanol and 200 µL of NaOH (40% *w*/*v*) under MW, as described above. The compound has been previously synthesized [53]. A different method has been reported. Yield: 71%; R_f_ = (hexane:acetone, 1:1): 0.6; m.p.: 130–133 °C; IR (Nujol, cm^−1^): 1670; ^1^H NMR (500 MHz, CDCI_3_) δ (ppm): 7.57 (dd, *J* = 16.3), 7.7 (d, *J* = 7.6 Hz, 1H), 7.49 (d, *J* = 7.6 Hz, 1H). ^13^C NMR (125 MHz, CDCI_3_) δ (ppm): 185.6 (C=O), 135.1, 134.8, 133.6, 132.0, 131.7, 130.0, 128.7, 127.4, 126.9, 126.5, 125.0, 124.7, 68.9 (C-O-C); Anal. C, H. Elemental Analysis: Expected % (C_27_H_20_O_2_): C, 86.14; H, 5.36; Found %: C, 86.04; H, 5.56; *m*/*z* (ESI positive) 399.4 [M + Na]^+^. The spectral data were in agreement with the literature data.

*(3Z,5Z)-3,5-bis(naphthalen-1-ylmethylene)dihydro-2H-thiopyran-4(3H)-one* (**1n**): According to the general method B. Tetrahydro-*4H*-thiopyran-4-one and naphthyl-aldehyde-1 were used at a molar ratio of 1:2, in 3 mL ethanol and 200 µL NaOH (40% *w*/*v*) under MW, as described above. The compound has been previously synthesized [53]. A different method has been reported. Yield: 50%; R_f_ (CH_3_COOCH_3_:petroleum ether, 1:1): 0.46; m.p.: 101–104 °C;IR (Nujol, cm^−1^): 1650; ^1^H NMR (500 MHz, CDCI_3_) δ (ppm): 8.06–7.99 (m, 2H), 7.42–7.45 (m, 2H), 7.69–7.93 (m, 12H), 4.59–4.62 (br, 4H).^13^C NMR (125 MHz, CDCI_3_) δ (ppm): 188.7 (C=O), 137.6–137.6 (m), 135.9 (s), 135.4 (s), 133.4 (s), 132.3–132.3 (m), 131.7–131.7 (m), 129.2 (s), 128.5 (s), 126.9 (s), 126.6 (s), 126.3 (s), 124.9 (s), 124.7 (s), 30.4 (C-S-C); Anal. C, H. Elemental Analysis: Expected % (C_27_H_20_OS): C, 82.62; H, 5.14; Found %: C, 82.52; H, 5.38; *m*/*z* (ESI positive) 424.5 [M + CH_3_OH]^+^. The spectral data were in agreement with the literature data.

*(3E,5E)-3,5-dibenzylidenepiperidine-4-thione* (**1o**): According to general method C, as it was described above. Yield: 71%; R_f_ (CH_3_COOCH_3_:petroleum ether, 1:1) 0.44; m.p.: semisolid; ^1^H-NMR (300 MHz, DMSO-*d*_6_) δ (ppm): 4.70–4.80 (s, 4H), 7.33 (s, 1H), 7.48 (d, *J* = 8.1 Hz, 1H), 7.50–7.61 (m, 8H), 7.79–7.80 (br, 2H); The NH absorption of piperidine-4-thione was not observed in the ^1^H NMR spectra of the compound as it has been refined for the NH absorption of piperidinone. ^13^C-NMR (75 MHz, DMSO-*d*_6_) δ (ppm): 232 (C=S), 135.2, 131.2, 128.5, 128.6, 128, 120.4, 51.7. Anal. C, H, N. Elemental Analysis: Expected %(C_19_H_17_NS): C, 78.31; H, 5.88; N, 4.81; Found %: C, 78.01; H, 5.69; N, 4.78.

*(3E,5E)-3,5-bis(naphthalen-1-ylmethylene)piperidine-4-thione* (**1p**): According to general method C, as it was described above. Yield: 37%; R_f_ (CH_3_COOCH_3_:petroleum ether, 1:1) 0,4; m.p.: semisolid; ^1^H-NMR (300 MHz) DMSO-*d*_6_) δ (ppm): 4.36–4.43 (s, 4H), 7.30–7.38 (m, 2H), 7.50–7.59 (m, 6H), 7.61–7.79 (m, 2H), 7.83–7.99 (m, 6H); The NH absorption of piperidine-4-thione was not observed in the ^1^H NMR spectra of the compound as it has refined for the NH absorption of piperidinone. ^13^C-NMR (75 MHz) δ (ppm): 234 (C=S), 137.2, 136.0, 135.6, 135.2, 134.5, 133.2, 132.5, 131.4, 131.2, 130.9, 128.5, 128.6, 128, 127.7, 126.3, 126.0, 124.0, 122.9, 120.4, 52.7; Anal. C, H, N. Elemental Analysis: Expected %(C_27_H_21_NS): C, 82.83; H, 5.41; N, 3.58; Found %: C, 82.98; H, 5.54; N, 3.69.

*(3E,5E)-3,5-bis(benzofuran-2-ylmethylene)piperidin-4-one* (**1q**): According to general method B, 4-piperidinone hydrochloride monohydrate (2.72 × 10^−3^ mol) and benzofuryl-aldehyde-2 (2 × 2.72 × 10^−3^ mol) were used at a molar ratio of 1:2 in 3 mL of ethanol and 200 µL of NaOH (40% *w*/*v*) under MW, as described above. Yield: 62%; R_f_ (CH_3_COOCH_3_:petroleum ether, 1:1) 0.5; m.p.: 101–104 °C; ^1^H-NMR (500 MHz, CDCl_3_) δ (ppm): 4.22–4.36 (s, 4H), 7.41–7.59 (m, 7H), 7.62–7.86 (m, 5H). The NH absorption spectra of piperidone were not observed in the NMR spectra of the compounds because it has already been refined by other researchers [43]. ^13^C-NMR (125 MHz) δ (ppm): 41.3, 43.7, 106,8, 111,5, 120.5, 120.9 124.0, 125.6,127.9, 129.8, 129.9, 130.0, 130.1, 131.0, 131.9, 132.0, 133.8, 133.9, 135.6, 142.5, 156.9, 157.8, 189 (C=O), Anal. C, H, N. Elemental Analysis: Expected % (C_23_H_17_NO3): C, 77.73; H, 4.82; N, 3.94; Found %: C, 77.87; H,4.98; N, 3.78.

#### 3.2.2. Synthesis of Acids **2a**–**c** [37]

##### General Method D—Synthetic Method for the Synthesis of Hybrids of Curcumin Analogues **3a**–**3h**

(**1g** and **1j**) (1 eq.) with acids (**2a**–**c**) (1.5 eq.), HOBT (2 eq.) and EDCI.HCL (3.5 eq.) were dissolved in dry DMF (3–5 mL). The mixture was stirred at room temperature for 10 min up to 24 h under nitrogen or argon gas. After the completion of the reaction (TLC monitoring), the mixture was washed with distilled water and cooled to room temperature. The precipitated products **3a**–**3h** were collected by filtration and washed with water. The crude products were recrystallized from 95% aqueous ethanol.

*(3E,5E)-1-cinnamoyl-3,5-bis(naphthalen-1-ylmethylene)piperidin-4-one* (**3a**): The synthesis followed general method D. The crude product was recrystallized from 95% aqueous ethanol. Yield: 24%; R_f_ (CH_3_COOCH_3_:petroleum ether, 1:1) 0.62; m.p.: 65–68 °C; ^1^H NMR (500 MHz, CDCl_3_) δ (ppm): 7.90 (dd, *J* = 21.5, 11.7 Hz, 1H), 7.72 (d, *J* = 7.1 Hz, 1H), 7.63–7.27 (m, 2H), 7.23–6.77 (m, 1H), 6.64 (d, *J* = 7.5 Hz, 1H), 6.07 (d, *J* = 15.4 Hz, 1H), 4.81 (d, *J* = 122.8 Hz, 1H); ^13^C NMR (125 MHz, CDCl_3_) δ (ppm): 187.3 (C=O on the ring), 165.9 (Ν-C=O), 143.6, 134.7, 133.9, 130.2, 129.7, 128.9, 128.7, 127.6, 127.4, 125.4, 116.3, 29.9 (C-N-C); Anal. C, H, N. Elemental Analysis: Expected (C_36_H_27_NO_2_): C, 85.52; H, 5.38; N, 2.77; Found: C, 85.64; H, 5.42; N, 2.97; *m*/*z* (ESI positive) 537.6 [M + CH_3_OH]^+^.

*(3E,5E)-3,5-dibenzylidene-1-((E)-3-(naphthalen-1-yl)acryloyl)piperidin-4-one* (**3b**): The synthesis followed general method D. The crude product was recrystallized from 95% aqueous ethanol. Yield: 64%; R_f_ (CH_3_COOCH_3_:petroleum ether, 1:1): 0.67; m.p.: 210–211°C; ^1^H NMR (500 MHz, CDCl_3_) δ (ppm): 8.36 (d, *J* = 15.2 Hz, 1H), 8.11–8.07 (m, 1H), 7.88 (s, 1H), 7.82 (t, *J* = 7.8 Hz, 1H), 7.70–7.26 (m, 3H), 7.06 (d, *J* = 7.1 Hz, 1H), 6.51 (d, *J* = 15.2 Hz, 1H), 4.98 (d, *J* = 66.7 Hz, 1H); ^13^C NMR (125 MHz, CDCl_3_) δ (ppm): 187.3 (C=O on the ring), 165.8 (Ν-C=O), 140.6, 134.7, 133.6, 132.4, 131.4, 130.1, 129.7, 129.0, 128.6, 126.8, 126.2, 125.4, 124.4, 123.6, 119.2 (C-N-C); Anal. C, H, N. Elemental Analysis: Expected (C_32_H_25_NO_2_): C, 84.37; H, 5.53; N, 3.07; Found: C, 84.30; H, 5.35; N, 3.12; *m*/*z* (ESI positive) 487.5 [M + CH_3_OH]^+^.

*(3E,5E)-1-((E)-3-(naphthalen-1-yl)acryloyl)-3,5-bis(naphthalen-1-ylmethylene)piperidin-4-one* (**3c**): The synthesis followed general method D. The crude product was recrystallized from 95% aqueous ethanol. Yield: 18%; R_f_ (CH_3_COOCH_3_:petroleum ether, 1:1): 0.64; m.p.: 154–156 °C; ^1^H NMR (500 MHz, CDCl_3_) δ (ppm):8.49 (s, 1H), 8.24 (d, *J* = 15.2 Hz, 1H), 8.11–7.81 (m, 2H), 7.80–7.70 (m, 1H), 7.63–7.29 (m, 2H), 7.06–7.01 (m, 1H), 6.47 (d, *J* = 7.2 Hz, 1H), 6.20 (d, *J* = 15.1 Hz, 1H), 4.84 (d, *J* = 115.4 Hz, 1H); ^13^C NMR (125 MHz, CDCl_3_) δ (ppm): 186.9 (C=O on the ring), 165.5 (Ν-C=O), 140.4, 133.5, 133.3, 131.8, 131.1, 129.8 (d, *J* = 26.0 Hz), 128.5 (d, *J* = 26.6 Hz), 127.2–125.8 (m), 125.1, 124.4, 123.8, 123.2,118.52 (C-N-C); Anal. C, H, N. Elemental Analysis: Expected (C_40_H_29_NO_2_): C, 86.46; H, 5.26; N, 2.52; Found: C, 86.41; H, 5.25; N, 2.50; *m*/*z* (ESI positive) 578.6 [M + Na]^+^.

*(3E,5E)-1-cinnamoyl-3,5-bis(4-fluorobenzylidene)piperidin-4-one* (**3d**): The synthesis followed general method D. The crude product was recrystallized from 95% aqueous ethanol. Yield: 9%; R_f_ (CH_3_COOCH_3_:petroleum ether,1:1): 0.9; m.p.: 178–180 °C; ^1^H NMR (500 MHz, CDCl_3_) δ (ppm): 7.81 (s, 1H), 7.72 (s, 1H), 7.49 (dd, *J* = 21.9, 11.0 Hz, 1H), 7.30 (dd, *J* = 15.3, 7.3 Hz, 1H), 7.18 (t, *J* = 8.2 Hz, 1H), 7.09 (d, *J* = 7.1 Hz, 1H), 6.38 (d, *J* = 15.4 Hz, 1H), 4.88 (s, 1H); ^13^C NMR (125 MHz, CDCl_3_) (ppm): 187.18 (C=O on the ring), 166.01 (Ν-C=O), 163.56 (d, *J* = 252.0 Hz, C-F), 143.88, 134.86, 133.16–132.05 (m), 131.06–130.93 (m), 130.18, 128.98, 127.84, 116.40 (d, *J* = 22.0 Hz), 29.95 (C-N-C); Anal. C, H, N. Elemental Analysis: Expected (C_28_H_21_F_2_NO_2_): C, 76.18; H, 4.79; F, 8.61; N, 3.17; O, 7.25; *m*/*z* (ESI positive) 596.4 [M + CH_3_OH + Na]^+^.

*(3E,5E)-1-((E)-3-(naphthalen-1-yl)acryloyl)-3,5-bis(thiophen-2-ylmethylene)piperidin-4-one* (**3e**): The synthesis followed general method D. The crude product was recrystallized from 95% aqueous ethanol. Yield: 55%; R_f_ (CH_3_COOCH_3_:petroleum ether, 1:1): 0.8; m.p.: 199–203 °C; ^1^H NMR (500 MHz, CDCl_3_) δ (ppm):8.50 (d, *J* = 15.2 Hz, 1H), 8.18–8.14 (m, 1H), 8.04 (s, 1H), 7.87–7.83 (m, 1H), 7.62 (s, 1H), 7.54–7.38 (m, 2H), 7.20 (dd, *J* = 5.0, 3.8 Hz, 1H), 6.97 (d, *J* = 15.2 Hz, 1H), 5.07 (s, 1H); ^13^C NMR (125 MHz, CDCl_3_) δ (ppm):185.6 (C=O on the ring), 165.7 (Ν-C=O), 141.0, 134.0, 133.5, 132.5, 131.3, 129.9, 128.5 (d, *J* = 7.8 Hz), 126.7, 126.1, 125.3, 124.5, 123.5, 119.2 (C-N); Anal. C, H, N. Elemental Analysis: Expected (C_28_H_21_NO_2_S_2_): C, 71.92; H, 4.53; N, 3.00; Found: C, 71.89; H, 4.55; N, 3.28; *m*/*z* (ESI positive) 522.6 [M + CH_3_OH + Na]^+^.

*(3E,5E)-3,5-bis(4-fluorobenzylidene)-1-((E)-3-(naphthalen-1-yl)acryloyl)piperidin-4-one* (**3f**): The synthesis followed general method D. The crude product was recrystallized from 95% aqueous ethanol. Yield: 9%; R_f_ (CH_3_COOCH_3_:petroleum ether, 1:1): 0.68; m.p.: 191–194 °C; ^1^H NMR (500 MHz, CDCl_3_) δ (ppm): 8.36 (d, *J* = 15.2 Hz, 1H), 8.09 (dd, *J* = 8.3, 5.0 Hz, 1H), 7.95–7.78 (m, 1H), 7.61–7.31 (m, 2H), 7.19–7.08 (m, 1H), 4.94 (d, *J* = 62.3 Hz, 1H); ^13^C NMR (125 MHz, CDCl_3_) δ (ppm): 186.8 (C=O on the ring), 165.7 (Ν-C=O), 163.22 (d, *J* = 251.5 Hz) C-F, 140.8, 133.5, 132.1, 131.2, 130.1, 128.7, 128.5, 126.9, 126.7, 126.1, 125.4, 125.2, 125.1, 124.2, 123.4, 123.2, 118.9, 116.1, 115.9, 29.6 (C-N-C); Anal. C, H, N. Elemental Analysis: Expected (C_32_H_23_F_2_NO_2_): C, 78.19; H, 4.72; N, 2.85; Found: C, 78.15; H, 4.92; N, 2.78; *m*/*z* (ESI positive) 546.5 [M + CH_3_OH + Na]^+^.

*(3E,5E)-3,5-dibenzylidene-1-cinnamoylpiperidin-4-one* (**3g**) [51]: The synthesis followed general method D. The crude product was recrystallized from 95% aqueous ethanol. Yield: 72%; R_f_ (CH_3_COOCH_3_:petroleum ether, 1:1): 0.83; m.p.: 188–190 °C; ^1^H NMR (500 MHz, CDCl_3_) δ (ppm): 7.86 (s, 1H), 7.53–7.43 (m, 2H), 7.38–7.27 (m, 1H), 7.23 (t, *J* = 7.4 Hz, 1H), 7.04 (d, *J* = 7.3 Hz, 1H), 6.37 (d, *J* = 15.4 Hz, 1H), 4.90 (dd, *J* = 46.4, 32.4 Hz, 1H); ^13^C NMR (125 MHz, CDCl_3_) δ (ppm): 187.4 (C=O on the ring), 165.9 (Ν-C=O), 143.6, 134.8, 134.7, 129.9, 129.7, 129.5, 128.8, 127.8, 116.4 (C-N). Anal. C, H, N. Elemental Analysis: Expected (C_28_H_23_NO_2_): C, 82.94; H, 5.72; N, 3.45; Found: C, 82.78; H, 5.92; N, 3.78; *m*/*z* (ESI positive) 428.5 [M + Na]^+^.

*(3E,5E)-1-((E)-3-(3,4-dihydroxyphenyl)acryloyl)-3,5-bis(naphthalen-1-ylmethylene)piperidin-4-one* (**3h**): A modified synthetic procedure was followed. The cinnamoyl chloride of acid **2a** (0.003 mol), thionyl chloride (0.007 mol), *N*,*N*-dimethylformamide (0.04 mL), and dry dichloroethane (40 mL) were heated under reflux for 6 h. The isolated chloride (after in vacuo evaporation) was dissolved in dichloroethane (30 mL). To this solution, curcumin analogue **1g** (0.003 mol) was added, and the reaction mixture was cooled in an ice-bath for 20 min. A solution of Et_3_N (0.006 mol) in dichloroethane (20 mL) was added dropwise over 0.5 h. The reaction mixture was cooled in an ice-bath that was removed after the addition and was led gradually to r.t. The mixture was stirred for 6 h after the addition of Et_3_N. The solvent was evaporated under reduced pressure. A solution of HCl (2% *w*/*v*, 30 mL) was added and the material was stirred for 1 h at r.t. The precipitate was collected, washed with water (3 × 50 mL) and MeOH (30 mL), and dried. The hybrid was recrystallized from a mixture of ethanol and water (3:1). The crude product was recrystallized from 95% aqueous ethanol. Yield: 39%; R_f_ (CH_3_COOCH_3_:petroleum ether, 1:1): 0.69; m.p.: 152–153 °C; ^1^H NMR (500 MHz, CDCl_3_) δ (ppm): 8.49 (s, 1H), 7.97 (d, *J* = 45.5 Hz, 1H), 7.45 (d, *J* = 94.5 Hz, 2H), 6.85 (s, 1H), 6.34 (s, 1H), 6.03 (d, *J* = 14.5 Hz, 1H), 4.97–4.52 (m, 1H); ^13^C NMR (125 MHz, CDCl_3_) δ (ppm):186.6 (C=O on the ring), 167.5 (Ν-C=O), 142.4 (C-OH), 141.0 (C-OH), 133.2, 128.3, 124.8, 124.4, 124.2, 123.1, 122.5, 116.8, 29.3 (C-N-C); Anal. C, H, N. Elemental Analysis: Expected (C_36_H_27_NO_4_): C, 80.43; H, 5.06; N, 2.61; Found: C, 80.28; H, 5.03; N, 2.91; *m*/*z* (ESI positive) 560.6 [M + Na]^+^.

### 3.3. Physicochemical Studies

#### Determination of R_M_ Values

Reversed phase TLC (RPTLC) was performed on silica gel plates impregnated with 55% (*v*/*v*) liquid paraffin in light petroleum ether. The mobile phase was a methanol/water mixture (77/23, *v*/*v*). The plates were developed in closed chromatography tanks saturated with the mobile phase at 24 °C. Spots were detected under UV light. R_M_ values were determined from the corresponding R_f_ values (from five individual measurements) using the equation R_M_ = log[(1/R_f_) − 1]; Table 2 [30].

### 3.4. Biological In Vitro Assays

The in vitro assays were performed at a concentration of 100 µM (a 10 mM stock solution in DMSO was used, from which several dilutions were made for the determination of IC_50_ values), at least in triplicate, and the standard deviation of absorbance was less than 10% of the mean. The compounds were diluted in 0.1% DMSO under sonification in an appropriate buffer in several dilutions (Table 3). Statistical comparisons were made using the Student T-test. A statistically significant difference was defined as *p* < 0.05.

#### 3.4.1. Inhibition of Linoleic Acid Peroxidation

In vitro study was evaluated as reported previously by our group [77]. Ten microliters of the 16 mM sodium linoleate solution were added to the UV cuvette containing 0.93 mL of a 0.05 M phosphate buffer, pH 7.4, prethermostated at 37 °C. The oxidation reaction was initiated at 37 °C under air by the addition of 50 µL of a 40 mM AAPH solution, which was used as a free radical initiator. Oxidation was carried out in the presence of aliquots (10 µL) in the assay without antioxidants and monitored at 234 nm. Lipid oxidation was recorded in the presence of the same level of DMSO and served as a negative control. Trolox was used as the appropriate standard (positive control).

#### 3.4.2. Soybean Lipoxygenase Inhibition Study

The in vitro study was evaluated as reported previously by our group [28,30]. The tested compounds were incubated at room temperature with sodium linoleate (0.1 mM) and 0.2 mL of enzyme solution (1/9 × 104 *w*/*v* in saline). The method was based on the conversion of sodium linoleate to 13-hydroperoxylinoleic acid at 234 nm. Nor-dihydroguaeretic acid NDGA (IC_50_ = 0.45 µM) was used as a standard (positive control) (Table 3). In order to determine the IC_50_ values, different concentrations were used. A blank determination was used first to serve as a negative control. The results are given in Table 3.

#### 3.4.3. AChE Inhibition Study

The in vitro study was evaluated as reported previously by our group. A modified Ellman procedure was followed [81]. The assay was performed in a pH 8 phosphate buffer (0.1 M) and used the thiol ester acetylthioline (0.01 M), which was hydrolyzed by AChE (3.5 U/mL) to produce thiocholine and acetate. The thiocholine reduced DTNB (0.01 M in phosphate 0.1 M pH 7), thus liberating nitrobenzoate, which absorbed at 412 nm. A blank determination was used first to serve as a negative control. As a standard inhibitor, tacrine (positive control) was used.

#### 3.4.4. Cyclooxygenase 2 Inhibition Study

The in vitro study of cyclooxygenase (COX) activity [82] was determined by using arachidonic acid (AA) as the substrate and *N*,*N*,*N*,*N*-tetramethylphenylenediamine (TMPD) as the co-substrate. The reaction mixture (1 mL) contained 0.75 mM heme, 128 mM TMPD, 80 mM AA, and 1.5 mg enzyme in 0.1 M Tris/HCl (pH 8.5). The oxidation of the substrate was measured at room temperature by monitoring the increase of absorbance at 611 nm. The absorption due to the spontaneous oxidation of TMPD was subtracted from the initial rate of oxidation observed in the presence of AA. The inhibition of the compounds was determined after preincubation for 6 min with the enzyme in the presence of heme and TMPD, and the reaction was started by adding AA. A blank determination was used first to serve as a negative control. Indomethacin was used as a reference COX-2 inhibitor (positive control) (Table 3).

### 3.5. Computational Methods.

#### 3.5.1. Molecular Docking Studies on Soybean Lipoxygenase

UCSF Chimera was used for the visualization of the protein (PDB code: 3PZW) [83]. Water molecules were removed, missing residues were added with Modeller [84], hydrogen atoms and AMBER99SB-ILDN charges were added, and the charge on iron was set to +2.0 with no restraint applied to the iron atom and the ligands. Open Babel was used to generate and minimize ligand 3D coordinates using the MMFF94 force field [85]. Ligand topologies and parameters were generated by ACPYPE (AnteChamber PYthon Parser interfacE) [86] using AnteChamber [87]. Energy minimizations were carried out using the AMBER99SB-ILDN force field with GROMACS 4.6. Docking was performed with AutoDock Vina (1.1.2) [88] by applying a grid box of size 100, 70, AND 70 Å in the X, Y, and Z dimensions, respectively. The generation of docking input files and the analysis of the docking results were accomplished with UCSF-Chimera. Docking was carried out with an exhaustiveness value of 10 and a maximum output of 20 docking modes.

#### 3.5.2. Molecular Docking Studies on AChE

Until today, there had been no experimental 3D-structure of *Electrophorus electricus* AChE in the Protein Data Bank. The UniProt entry for *E. electricus* AChE is O42275 [89]. Using the amino acids sequence annotated in UniProt, a 3D-model was generated with I-TASSER [90,91,92]. The UCSF Chimera MatchMaker procedure was used for aligning the generated 3D-model of AChE with the following 3D-protein structures from the PDB—4BDT.A, 4EY5.A, 4EY6.A, and 4EY7.A, all of which contained active ligands used as drugs and minimized the necessity to consider protein flexibility [93]. The aligned PDB structures were scripted out of the protein part, and the ligands were maintained. Therefore, four new complexes were obtained from the *Electrophorus electricus* 3D-AChE with X-ray co-crystallized ligands: 4BDT:HUW, 4EY5:HUP, 4EY6:GNT, and 4EY7:E20. Finally, these four new complexes were geometrically optimized by minimization using UCSF-Chimera with the AMBER14SB force website field [94,95]. TIP3P [96] was used to describe the water model and total system charge neutralization with Na + Cl^−^ ions. Ligand topologies and parameters were generated with AnteChamber [97]. The 4 optimized complexes were used for the cross-docking simulations. Docking simulations were run with a Vinardo scoring function [98], as implemented in SMINA [99]. Docking was carried out using a grid box of size 25 Å in the X, Y, and Z dimensions and with an exhaustiveness value of 64 and a maximum output of 20 docking modes. Docking results analysis and visual inspection were carried out using UCFS Chimera. All ligands were docked to the 4 optimized complexes. The best docked pose was selected by considering the docking score.

#### 3.5.3. In Silico Determination of Drug-Likeness

Compounds were subjected to molecular properties prediction in the online software versions of: (i) Molinspiration software version 2016.10 (www.molinspiration.com) [59], (ii) MolSoft platform (http://molsoft.com/mprop/) [61,95], (iii) CypRules platform (https://cyprules.cmdm.tw/) [62], (iv) preADMET platform (https://preadmet.bmdrc.kr/) [63], and (v) SwissADME platform http://www.swissadme.ch/index.php [64].

The CypRules server predicted the metabolizing cytochrome P450 (CYPs) inhibition, including that of CYP1A2, CYP2C19, CYP2C9, CYP2D6, and CYP3A4, and the results were based on the C5.0 algorithm. The rules were calculated on a basis of Mold2 2D descriptors.

## 4. Conclusions

**Figure 10:**
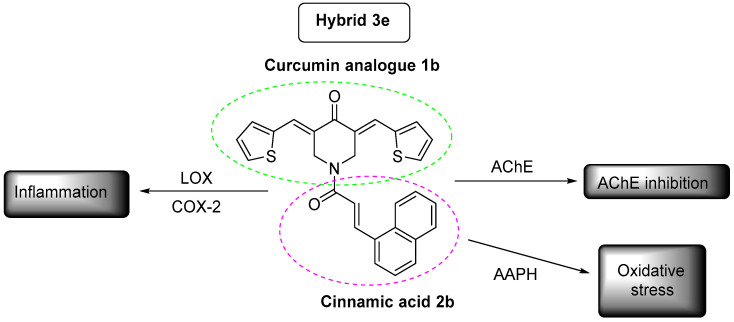
Hybrid 3e is presenting the most interesting profile.

The designed and synthesized curcumin analogues and hybrids of cinnamic acids presented multi-target activity against different targets. Hybrid **3e** (Figure 10) was the most significant pleiotropic derivative, in terms of activities. This hybrid will be used as a lead compound for further theoretical structural modifications. Hybrid **3a** also presented a promising pleiotropic profile.

Docking studies on SLOX showed hydrophobic interactions with the amino acids, whereas docking studies on AChE exhibited hydrophobic interactions and π-stacking interactions.

All hybrids could be easily transported, diffused, and absorbed through the BBB, thus presenting good oral bioavailability. No inhibition for CYP1A2, CYP2C9, CYP2C19, CYP2D6, and CYP3A4 was noticed. According to the Ames test, all the hybrids induced mutagenicity, with the exception of **3d**.

Further investigation is in progress to delineate the role of these hybrids on inflammation and Alzheimer’s disease.

## Figures and Tables

**Table 1:** Experimentally determined lipophilicity values (R_M_).

Compounds	R_M_ ± S.D.	Milog P ^a^
**1k**	0.872 ± 0.039	6.42
**1l**	0091 ± 0.036	3.91
**1m**	0.577 ± 0.027	5.87
**1n**	0.131 ± 0.142	6.41
**1o**	−0.848 ± 0.19	4.62
**1p**	−0.679 ± 0.035	6.58
**1q**	−0.019 ± 0.143	5.39
**2a**	−0.485 ± 0.044	1.91
**2b**	−0.576 ± 0.077	2.89
**2c**	−0.852 ± 0.059	0.94
**3a**	0.599 ± 0.024	5.88
**3b**	0.509 ± 0.062	7.58
**3c**	−0.658 ± 0.061	6.60
**3d**	0.237 ± 0.06	8.45
**3e**	0.514 ± 0.039	5.95
**3f**	0.564 ± 0.045	6.04
**3g**	0.235 ± 0.039	6.93
**3h**	−0.657 ± 0.013	5.62
**Curcumin**	0.175 ± 0.029	2.30

SD < 10%; ^a^ theoretically calculated logarithm of partition coefficient between n-octanol and water (www.molinspiration.com [59]).

**Table 2:** Molecular properties prediction—Lipinski’s Rule of Five.

Compounds	Milog P ^a^	TPSA ^b^	N^o^ Atoms	N^o^O,N ^c^	N^o^ OH, NH ^d^	N^o^ Violations	N^o^ Rotational Bonds ^e^	Volume ^f^	MW ^g^	logBB ^h^
**1k**	6.42	17.07	28	1	0	1	2	340.01	360.46	0.660
**1l**	3.91	26.30	21	2	0	0	2	261.02	276.33	0.179
**1m**	5.87	26.30	29	2	0	1	2	349.00	376.45	0.483
**1n**	6.41	17.07	29	1	0	1	2	358.14	392.52	0.659
**1o**	4.62	12.03	21	1	1	0	2	273.31	291.42	0.760
**1p**	6.58	12.03	29	1	1	1	2	361.30	391.54	1.064
**1q**	5.39	38.31	27	3	1	1	2	324.43	371.46	0.616
**2a**	1.91	37.30	11	2	1	0	2	138.46	148.16	0.087
**2b**	2.89	37.30	15	2	1	0	2	182.45	198.22	0.239
**2c**	0.94	77.75	13	4	3	0	2	154.50	180.16	−0,468
**3a**	5.88	37.38	33	3	0	1	3	399.51	429.52	0.702
**3b**	7.58	37.38	39	3	0	2	4	470.61	505.62	0.637
**3c**	6.60	37.38	35	3	0	1	4	426.62	455.56	0.485
**3d**	8.45	37.38	43	3	0	2	4	514.60	555.68	0.772
**3e**	5.95	37.38	33	3	0	1	4	392.49	441.48	0.384
**3f**	6.04	37.38	33	3	0	1	4	408.04	467.62	0.398
**3g**	6.93	37.38	37	3	0	1	4	436.48	491.54	0.536
**3h**	5.62	37.38	31	3	0	1	4	382.62	405.50	0.384
**Curcumin**	2.30	93.07	27	6	2	0	8	332.18	368.38	−0.410

^a^ Logarithm of partition coefficient between n-octanol and water (milog *P*); ^b^ topological polar surface area (TPSA); ^c^ number of hydrogen bond acceptors (n-ON); ^d^ number of hydrogen bond donors (n-OHNH); ^e^ number of rotatable bonds (n-rotb); ^f^ molecular volume; ^g^ molecular weight; and ^h^ blood–brain barrier.

**Table 3:** Prediction of metabolism (CYP) and excretion using the CypRules rids present mutagenicity in the Ames test and the SwissADME platforms.

Comp.	CYP1A2		CYP2C19				CYP3A4			
	CypRules	Swiss ADME	CypRules	Swiss ADME	CypRules	Swiss ADME	CypRules	Swiss ADME	CypRules	Swiss ADME
**1a**	No	No	No	No	No	No	No	No	No	No
**1b**	No	Inh.	No	Inh.	No	Inh.	No	No	No	Inh.
**1c**	No	No	No	Inh.	No	Inh.	No	No	No	No
**1d**	No	Inh.	No	No	No	No	No	No	No	Inh.
**1e**	No	No	No	No	No	No	No	Inh.	No	No
**1f**	No	Inh.	No	No	No	No	No	No	No	No
**1g**	No	No	No	No	No	Inh.	No	Inh.	No	No
**1h**	No	Inh.	No	Inh.	No	Inh.	No	No	No	Inh.
**1i**	No	Inh.	No	Inh.	No	Inh.	No	No	No	Inh.
**1j**	No	Inh.	No	No	No	Inh.	No	Inh.	No	No
**2a**	No	No	No	No	No	No	No	No	No	No
**2b**	No	No	No	No	No	No	No	No	No	No
**2c**	No	No	No	No	No	No	No	No	No	No
**3a**	No	No	No	No	No	No	No	No	No	Inh.
**3b**	No	No	No	No	No	No	No	No	No	Inh.
**3c**	No	No	No	No	No	No	No	No	No	No
**3d**	No	No	No	Inh.	No	Inh.	No	No	No	Inh.
**3e**	No	No	No	Inh.	No	No	No	No	No	Inh.
**3f**	No	No	No	No	No	No	No	No	No	Inh.
**3g**	No	No	No	Inh.	No	Inh.	No	No	No	Inh.
**3h**	No	No	No	No	No	No	No	No	No	No
**1k**	No	No	No	No	No	No	No	No	No	No
**1l**	No	No	No	Inh.	No	Inh.	No	Inh.	No	No
**1m**	No	No	No	No	No	No	No	No	No	No
**1n**	No	No	No	No	No	No	No	No	No	Inh.
**1o**	No	No	No	Inh.	No	Inh.	No	No	No	Inh.
**1p**	No	No	No	No	No	No	No	No	No	Inh.
**1q**	No	No	No	Inh.	No	Inh.	No	No	No	Inh.
**Curcumin (Keto)**	No	No	No	Inh.	No	No	No	No	No	Inh.
**Curcumin (Enol)**	No	No	No	Inh.	No	No	No	No	No	Inh.

**Table 4:** Toxicity assessment of the synthesized compounds calculated by the preADMET platform (https://preadmet.bmdrc.kr/).

	Ames test					Carcinogenicity	
Comp.	Ames Test	TA100_10RLI	TA100_NA	TA1535_10RLI	TA1535_NA	Carcino_Mouse	Carcino_Rat
**1a**	Mutagen	Negative	Negative	Negative	Negative	Positive	Negative
**1b**	Mutagen	Negative	Positive	Positive	Positive	Negative	Negative
**1c**	Mutagen	Negative	Negative	Negative	Positive	Positive	Positive
**1d**	Mutagen	Negative	Negative	Negative	Negative	Positive	Negative
**1e**	Mutagen	Negative	Negative	Negative	Negative	Negative	Positive
**1f**	Mutagen	Negative	Positive	Negative	Positive	Negative	Negative
**1g**	Mutagen	Negative	Positive	Negative	Positive	Negative	Negative
**1h**	Mutagen	Negative	Positive	Positive	Positive	Negative	Negative
**1i**	Mutagen	Negative	Positive	Negative	Positive	Negative	Negative
**1j**	Mutagen	Negative	Negative	Positive	Negative	Negative	Negative
**2a**	Mutagen	Positive	Negative	Negative	Negative	Negative	Negative
**2b**	Mutagen	Positive	Negative	Positive	Negative	Negative	Negative
**2c**	Mutagen	Negative	Positive	Negative	Positive	Negative	Positive
**3a**	Mutagen	Positive	Negative	Negative	Negative	Negative	Positive
**3b**	Mutagen	Positive	Negative	Negative	Negative	Negative	Positive
**3c**	Mutagen	Positive	Negative	Negative	Negative	Negative	Positive
**3d**	Non-Mutagen	Negative	Negative	Negative	Negative	Positive	Negative
**3e**	Mutagen	Positive	Negative	Negative	Negative	Negative	Negative
**3f**	Mutagen	Negative	Negative	Negative	Negative	Negative	Negative
**3g**	Mutagen	Positive	Negative	Positive	Negative	Negative	Positive
**3h**	Mutagen	Negative	Negative	Negative	Negative	Negative	Negative
**1k**	Mutagen	Positive	Negative	Negative	Negative	Negative	Negative
**1l**	Mutagen	Positive	Positive	Positive	Negative	Negative	Negative
**1m**	Mutagen	Positive	Negative	Negative	Negative	Positive	Negative
**1n**	Mutagen	Positive	Negative	Negative	Negative	Negative	Negative
**1o**	Mutagen	Negative	Positive	Negative	Positive	Negative	Negative
**1p**	Mutagen	Negative	Negative	Negative	Negative	Negative	Negative
**1q**	Mutagen	Negative	Positive	Negative	Negative	Positive	Positive
**Curcumin (Keto)**	Non-Mutagen	Negative	Negative	Negative	Negative	Negative	Positive
**Curcumin (Enol)**	Mutagen	Negative	Negative	Negative	Negative	Positive	Positive

**Table 5:** Multiple estimations of drug-likeness.

Drug-likeness						
Comp.	Lipinski	Ghose	Veber	Egan	Muegge	Bioavailability Score
**1a**	Yes *	Yes	Yes	Yes	No *	0.55
**1b**	Yes	Yes	Yes	Yes	Yes	0.55
**1c**	Yes	Yes	Yes	Yes	Yes	0.55
**1d**	Yes	Yes	Yes	Yes	Yes	0.55
**1e**	No **	No ***	Yes	No *	No **	0.17
**1f**	Yes	Yes	Yes	Yes	Yes	0.55
**1g**	Yes	Yes	Yes	Yes	Yes	0.55
**1h**	Yes	Yes	Yes	Yes	Yes	0.55
**1i**	Yes	Yes	Yes	Yes	Yes	0.55
**1j**	Yes	Yes	Yes	Yes	Yes	0.55
**2a**	Yes	No **	Yes	Yes	No *	0.56
**2b**	Yes	Yes	Yes	Yes	No *	0.56
**2c**	Yes	Yes	Yes	Yes	No *	0.56
**3a**	No **	No ***	Yes	No *	No *	0.17
**3b**	Yes *	No **	Yes	Yes	No *	0.55
**3c**	No **	No ***	Yes	No *	No *	0.17
**3d**	Yes *	No *	Yes	Yes	No *	0.55
**3e**	Yes	No **	Yes	Yes	No *	0.55
**3f**	Yes *	No ***	Yes	No *	No *	0.55
**3g**	Yes	Yes	Yes	Yes	No *	0.55
**3h**	Yes *	No ***	Yes	No *	No *	0.55
**1k**	Yes *	No *	Yes	No *	No **	0.55
**1l**	Yes	Yes	Yes	Yes	Yes	0.55
**1m**	Yes *	No *	Yes	Yes	No *	0.55
**1n**	Yes *	No *	Yes	No *	No *	0.55
**1o**	Yes	Yes	Yes	Yes	Yes	0.55
**1p**	Yes *	No **	Yes	Yes	No *	0.55
**1q**	Yes	Yes	Yes	Yes	Yes	0.55
**Curcumin (Keto)**	Yes	Yes	Yes	Yes	Yes	0.55
**Curcumin (Enol)**	Yes	Yes	Yes	Yes	Yes	0.56

* one violation, ** two violations, and *** three violations.

**Table 6:** % Anti-lipid peroxidation (2,2′-azobis(2-amidinopropane) hydrochloride—AAPH) of in vitro inhibition of soybean lipoxygenase, bovine cyclooxygenase (COX)-2, and eel acetylcholinesterase (AChE).

	LOX IC_50_ (μM) or (% Inhibition at 100 μM) ± SEM	AAPH Inhibition (%, at 100 μΜ)	COX-2 IC_50_ (μM) or (% Inhibition at 100 μM) ± SEM	AChE IC_50_ (μM) or (% Inhibition at 100 μM) ± SEM
1a *	37 μM ± 0.8			
1b *	330 μM ± 6.5			
1c *	380 μM ± 5.3			
1d *	410 μM ± 4.2			
1f *	36% ± 0.9			
1g *	8% ± 0.2			
**1j**	25.3% ± 0.4	36.2 ± 1.8	n.a	2.3% ± 0.01
**1k**	14 μΜ ± 0.2	100 ± 2.2	n.a	36% ± 1.4
**1l**	10% ± 0.3	42 ± 1.5	n.a	25% ± 0.7
**1m**	50.5 μΜ ± 1.8	100 ± 2.8	n.a	32% ± 0.6
**1n**	57.5 μΜ ± 0.9	100 ± 2.5	n.a	18% ± 0.3
**1o**	10 μΜ ± 0.1	8 ± 0.01	n.a	n.a
**1p**	35 μΜ ± 1.5	100 ± 3.3	n.a	26.60% ± 0.9
**1q**	3% ± 0.0	13 ± 0.2	n.a	16.50% ± 0.4
**2a**	56 μΜ ± 1.6	78 ±1.6	n.a	4.6% ± 0.2
**2b**	27.5 μΜ ± 0.7	78 ± 0.7	n.a	n.a
**2c**	10 μΜ ± 0.6	80 ± 2.2	57.5 μΜ ± 1.1	14% ± 0.1
**3a**	50 μΜ ± 1.4	91 ± 1.9	n.a	85 μΜ ± 1.3
**3b**	55 μΜ ± 1.1	90 ± 2.8	n.a	17% ± 0.6
**3c**	37.5 μΜ ± 0.7	88 ± 2.0	n.a	29% ± 0.8
**3d**	63.5 μΜ ± 1.5	84 ± 1.7	n.a	31% ± 0.5
**3e**	52 μΜ ± 1.9	91 ± 2.5	19% ± 0.2	70 μΜ ± 3.1
**3f**	55 μΜ ± 1.3	90 ± 2.4	n.a	24% ± 1.2
**3g**	82 μΜ ± 2.2	53 ± 1.6	n.a	100 μΜ ± 2.5
**3h**	34 μΜ ± 0.7	93 ± 2.2	#	#
**Curcumin**	38% ± 0.4	78 ± 1.8	19% ± 0.4	34% ± 1.3
**NDGA**	0.45 μΜ ± 0.03			
**Trolox**		92 ± 2.1		
**Indomethacin**			1.12 μΜ ± 0.1	
**Tacrine**				98% ± 1.5/(0.03 μΜ ± 0.01)

SEM: standard error of the mean; # not tested; n.a.: no activity under the experimental conditions; * [40]; means within each column differ significantly (*p* < 0.05).

## Supplementary Materials

Table SI, Supplementary Material.

## Abbreviations

AD: Alzheimer’s disease; Aβ plaques: amyloid-β plaques; AβPP: amyloid-β precursor protein; AChE: acetylcholinesterase; Ach: acetylcholine; AA: arachidonic acid; AAPH: 2,2′-azinobis(2-amidinopropane) hydrochloride; BACE-1: β-secretase or beta-site amyloid precursor protein cleaving enzyme 1; BBB: blood–brain barrier; CAs: cinnamic acids; CNS: central nervous system; COX: cyclooxygenase; CYP: Cytochrome; GSK3β: glycogen synthase kinase 3 beta; IL-6: interleukin 6; LDL: oxidation of low-density lipoprotein; Leu: Leucine; LOX: lipoxygenase; MW: microwave; NF-Kb: nuclear factor kappa-light-chain-enhancer of activated B cells; NFTs: neurofibrillary tangles; Nor-dihydroguaeretic acid: NDGA; Non Steroidal Antiiflammatory Drugs: NSAIDs; Phe: Phenylalanine; QSAR: quantitative structure activity relationships; ROS: reactive oxygen species; TNF-α: tumor necrosis factor-α; TYR: Tyrosine; TRP: Tryptophane.

## References

[1] Attitudes to Dementia-World Alzheimer Report 2019. https://www.alz.co.uk/research/WorldAlzheimerReport2019.Pdf.

[2] Song J., Malampati S., Zeng Y., Durairajan S.S.K., Yang C., Tong B.C., Iyaswamy A., Shang W., Sreenivasmurthy S.G., Zhu Z. (2019). A Small Molecule Transcription Factor EB Activator Ameliorates beta-amyloid Precursor Protein and Tau Pathology in Alzheimer’s Disease Models. Aging Cell.

[3] Hulya A., İlhami G. (2020). Potent Acetylcholinesterase Inhibitors: Potential Drugs for Alzheimer’s Disease. Mini–Rev. Med. Chem..

[4] Brufani M., Filocamo L. (1997). Rational Design of New Acetylcholinesterase Inhibitors. Alzheimer Disease.

[5] Gao X.-H., Liu L.-B., Liu H.-R., Tang J.-J., Kang L., Wu H., Cui P., Yan J. (2017). Structure–activity Relationship Investigation of Benzamide and Picolinamide Derivatives Containing Dimethylamine Side Chain As Acetylcholinesterase Inhibitors. J. Enzym. Inhib. Med. Chem..

[6] Dhull D.K., Jindal A., Dhull R.K., Aggarwal S., Bhateja D., Padi S.S.V. (2011). Neuroprotective Effect of Cyclooxygenase Inhibitors in ICV-STZ Induced Sporadic Alzheimer’s Disease in Rats. J. Mol. Neurosci..

[7] Qin W.-P., Eho L., Pompl P.N., Peng Y., Zhao Z., Xiang Z., Robakis N.K., Shioi J., Suh J., Pasinetti G.M. (2003). Cyclooxygenase (COX)-2 and COX-1 Potentiate β-Amyloid Peptide Generation through Mechanisms That Involve γ-Secretase Activity. J. Biol. Chem..

[8] Giannopoulos P.F., Joshi Y.B., Praticò D. (2013). Novel Lipid Signaling Pathways in Alzheimer’s Disease Pathogenesis. Biochem. Pharmacol..

[9] Firuzi O., Zhou J., Chinnici C.M., Wisniewski T., Praticò D. (2008). 5- Lipoxygenase Gene Disruption Reduces Amyloid-Beta Pathology in a Mouse Model of Alzheimer’s Disease. FASEB J..

[10] Joshi Y.B., Praticã D., Praticò D. (2015). The 5-Lipoxygenase Pathway: Oxidative and Inflammatory Contributions to the Alzheimer’s Disease Phenotype. Front. Cell. Neurosci..

[11] Shekhar S., Yadav S.K., Rai N., Kumar R., Tripathi M., Dey A.B. (2017). 5-LOX in Alzheimer’s Disease: Potential Serum Marker and In Vitro Evidences for Rescue of Neurotoxicity by Its Inhibitor YWCS. Mol. Neurobiol..

[12] Chainoglou E., Hadjipavlou-Litina D. (2020). Curcumin in Health and Diseases: Alzheimer’s Disease and Curcumin Analogues, Derivatives, and Hybrids. Int. J. Mol. Sci..

[13] Fang L., Gou S., Liu X., Cao F., Cheng L. (2014). Design, Synthesis and Anti-Alzheimer Properties of Dimethylaminomethyl-Substituted Curcumin Derivatives. Bioorganic Med. Chem. Lett..

[14] Venigalla M., Sonego S., Gyengési E., Sharman M.J., Münch G. (2016). Novel Promising Therapeutics Against Chronic Neuroinflammation and Neurodegeneration in Alzheimer’s Disease. Neurochem. Int..

[15] Swarit J., Ye H., Jurgen B. (2017). How Frequently Are Pan-Assay Interference Compounds Active? Large-Scale Analysis of Screening Data Reveals Diverse Activity Profiles, Low Global Hit Frequency, and Many Consistently Inactive Compounds. J. Med. Chem..

[16] Chainoglou E., Hadjipavlou-Litina D., Chainoglou E. (2019). Curcumin Analogues and Derivatives With Anti-Proliferative and Anti-Inflammatory Activity: Structural Characteristics and Molecular Targets. Expert Opin. Drug Discov..

[17] Burgos-Morón E., Calderón-Montaño J.M., Salvador J., Robles A., López-Lázaro M. (2010). The Dark Side of Curcumin. Int. J. Cancer.

[18] Liang G., Shao L., Wang Y., Zhao C., Chu Y., Xiao J., Zhao Y., Li X., Yang S. (2009). Exploration and Synthesis of Curcumin Analogues With Improved Structural Stability Both in Vitro and in Vivo As Cytotoxic Agents. Bioorganic Med. Chem..

[19] Shetty D., Kim Y.J., Shim H., Snyder J.P. (2014). Eliminating the Heart from the Curcumin Molecule: Monocarbonyl Curcumin Mimics (MACs). Molecumel.

[20] Yadav B., Taurin S., Rosengren R.J., Schumacher M., Diederich M., Somers-Edgar T.J., Larsen L. (2010). Synthesis and Cytotoxic Potential of Heterocyclic Cyclohexanone Analogues of Curcumin. Bioorganic Med. Chem..

[21] Das U., Sharma R., Dimmock J.R. (2009). 1,5-Diaryl-3-Oxo-1,4-Pentadienes: A Case for Antineoplastics With Multiple Targets. Curr. Med. Chem..

[22] Adams B.K., Ferstl E.M., Davis M.C., Herold M., Kurtkaya S., Camalier R.F., Hollingshead M.G., Kaur G., Sausville E.A., Rickles F.R. (2004). Synthesis and Biological Evaluation of Novel Curcumin Analogues As Anti-Cancer and Anti-Angiogenesis Agents. Bioorg. Med. Chem..

[23] Lagisetty P., Powell D.R., Awasthi V. (2009). Synthesis and Structural Determination of 3,5-bis(2-Fluorobenzylidene)-4-Piperidone Analogues of Curcumin. J. Mol. Struct..

[24] Lagisetty P., Vilekar P., Sahoo K., Anant S., Awasthi V. (2010). CLEFMA-An Antiproliferative Curcuminoid from Structure-Activity Relationship Studies on 3,5-bis(benzylidene)-4-Piperidones. Bioorg. Med. Chem..

[25] Jha A., Duffield K.M. (2007). 3,5-Bis(arylmethylene)-4-Piperidone Derivatives As Novel Anticancer Agents. Indian J. Chem..

[26] Makarov M.V., Leonova E.S., Rybalkina E.Y., Tongwa P., Khrustalev V.N., Timofeeva T.V., Odinets I.L. (2010). Synthesis, Characterization and structure–activity Relationship of Novel N-Phosphorylated E,E-3,5-bis(thienylidene)piperid-4-Ones. Eur. J. Med. Chem..

[27] Aboul-Fadl T., El-Shorbagi A.N., AHozien Z., Sarhan A.W. (2001). Investigation of Alkylating, Antineoplastic and Anti-HIV Potentials of the Chalcones: 2-(3-arylpropenoyl)benzimidazole and Their Corresponding N1-Methyl Derivatives. Boll. Chim. Farm..

[28] Pontiki E., Hadjipavlou-Litina D. (2006). Antioxidant and Anti-Inflammatory Activity of Aryl-Acetic and Hydroxamic Acids As Novel Lipoxygenase Inhibitors. Med. Chem..

[29] Pontiki E., Geromichalos G., Papageorgiou A., Hadjipavlou-Litina D. (2009). Anticancer Activity and Quantitative-Structure Activity Relationship (QSAR) Studies of a Series of Antioxidant/Anti-Inflammatory Aryl-Acetic and Hydroxamic Acids. Chem. Biol. Drug Des..

[30] Pontiki E., Hadjipavlou-Litina D. (2007). Synthesis and Pharmacochemical Evaluation of Novel Aryl-Acetic Acid Inhibitors of Lipoxygenase, Antioxidants, and Anti-Inflammatory Agents. Bioorganic Med. Chem..

[31] Naz S., Ahmad S., Rasool S.A., Sayeed S.A., Siddiqi R. (2006). Antibacterial Activity Directed Isolation of Compounds from Onosma Hispidum. Microbiol. Res..

[32] Nardini M., D’Aquino M., Tomassi G., Gentili V., Di Felice M., Scaccini C. (1995). Inhibition of Human Low-Density Lipoprotein Oxidation by Caffeic Acid and Other Hydroxycinnamic Acid Derivatives. Free. Radic. Biol. Med..

[33] Yasuko K., Tomohiro N., Sei-Itsu M., Ai-Na L., Yasuo F., Takashi T. (1984). Caffeic Acid Is a Selective Inhibitor for Leukotriene Biosynthesis. Biochim. Biophys. Acta (BBA)–Lipids Lipid Metab..

[34] Szwajgier D., Borowiec K., Pustelniak K. (2017). The Neuroprotective Effects of Phenolic Acids: Molecular Mechanism of Action. Nutrients.

[35] Zang L.-Y., Cosma G., Gardner H., Shi X., Castranova V., Vallyathan V. (2000). Effect of Antioxidant Protection by P-Coumaric Acid on Low-Density Lipoprotein Cholesterol Oxidation. Am. J. Physiol. Physiol..

[36] Gao X., Tang J., Liu H., Liu L., Kang L., Chen W. (2018). Structure–activity Relationship Investigation of Tertiary Amine Derivatives of Cinnamic Acid As Acetylcholinesterase and Butyrylcholinesterase Inhibitors: Compared With That of Phenylpropionic Acid, Sorbic Acid and Hexanoic Acid. J. Enzym. Inhib. Med. Chem..

[37] Peperidou A., Pontiki E., Hadjipavlou-Litina D., Voulgari E., Avgoustakis K. (2017). Multifunctional Cinnamic Acid Derivatives. Molecules.

[38] Pontiki E., Peperidou A., Fotopoulos I., Hadjipavlou-Litina D.J. (2019). Cinnamate Hybrids: A Unique Family of Compounds With Multiple Biological Activities. Curr. Pharm. Biotechnol..

[39] Noureddin S.A., El-Shishtawy R.M., Al-Footy K.O. (2019). Curcumin Analogues and Their Hybrid Molecules As Multifunctional Drugs. Eur. J. Med. Chem..

[40] Katsori A.-M., Chatzopoulou M., Dimas K., AKontogiorgis C., Patsilinakos A., Trangas T., Hadjipavloulitina D. (2011). Curcumin Analogues As Possible Anti-Proliferative & Anti-Inflammatory Agents. Eur. J. Med. Chem..

[41] Katsori A.-M., Palagani A., Bougarne N., Hadjipavlou-Litina D., Haegeman G., Berghe W.V. (2015). Inhibition of the NF-κB Signaling Pathway by a Novel Heterocyclic Curcumin Analogue. Molecules.

[42] Abdulrahman A., Raju K., Farzana B., Amir S., Hasnah O., Rusli I., Tan C., Brian S., Kellen M., Alaa N. (2014). Facile, Regio- and Diastereoselective Synthesis of Spiro-Pyrrolidine and Pyrrolizine Derivatives and Evaluation of Their Antiproliferative Activities. Molecules.

[43] Singaram K., Marimuthu D., Baskaran S., Ramaswamy V. (2016). Synthesis and Antimicrobial Activity of New 3,5-Diarylidene-4-Piperidone Derivatives. J. Serb. Chem. Soc..

[44] Wei X., Du Z., Zheng X., Cui X.-X., Conney A.H., Zhang K. (2012). Synthesis and Evaluation of Curcumin-Related Compounds for Anticancer Activity. Eur. J. Med. Chem..

[45] Nehmedo G., Fawzy Siva P., Walid F., El Sayed S., Aladdin S., Adel G. (2019). Synthesis, Human Topoisomerase IIa Inhibitory Properties and Molecular Modeling Studies of Antiproliferative Curcumin Mimics. RSC Adv..

[46] Xie X., Tu J., You H., Hu B. (2017). Design, Synthesis, and Biological Evaluation of Novel EF24 and EF31 Analogs As Potential IκB Kinase β Inhibitors for the Treatment of Pancreatic Cancer. Drug Des. Dev. Ther..

[47] Dimmock J., Padmanilayam M.P., Puthucode R.N., Nazarali A.J., Motaganahalli N.L., Zello G.A., Quail J.W., Oloo E.O., Kraatz H.-B., Prisciak J.S. (2001). A Conformational and Structure−Activity Relationship Study of Cytotoxic 3,5-Bis(arylidene)-4-Piperidones and RelatedN-Acryloyl Analogues. J. Med. Chem..

[48] Zhang W., Bai H., Han L., Zhang H., Xu B., Cui J., Wang X., Ge Z., Li R. (2018). Synthesis and Biological Evaluation of Curcumin Derivatives Modified With α-Amino Boronic Acid As Proteasome Inhibitors. Bioorganic Med. Chem. Lett..

[49] Almansour A.I., Suresh Kumar R., Arumugam N., Basiri A., Kia Y., Ashraf Ali M. (2015). An Expedient Synthesis, Acetylcholinesterase Inhibitory Activity, and Molecular Modeling Study of Highly Functionalized Hexahydro-1,6-Naphthyridines. BioMed Res. Int..

[50] Yalda K., Hasnah O., Raju K., Alireza B., Vikneswaran M. (2014). Synthesis and Discovery of Highly Functionalized Mono- and Bis-Spiro-Pyrrolidines As Potent Cholinesterase Enzyme Inhibitors. Bioorganic Med. Chem. Lett..

[51] Dimmock J., Jha A., Zello G.A., Quail J.W., Oloo E.O., Nienaber K.H., Kowalczyk E.S., Allen T.S., Santos C.L., De Clercq L. (2002). Cytotoxic N-[4-(3-Aryl-3-Oxo-1-propenyl)phenylcarbonyl]-3,5- bis(phenylmethylene)-4-Piperidones and Related Compounds. Eur. J. Med. Chem..

[52] Dimmock J.R., Arora V.K., Wonko S.L., Hamon N.W., Quail J.W., Jia Z., Warrington R.C., Fang W.D., Lee J.S. (1990). 3,5-Bis-Benzylidene-4-Piperidones and Related Compounds With High Activity towards P388 Leukemia Cells. Drug Des. Deliv..

[53] Hossain M., Das S., Das U., Doroudi A., Zhu J., Dimmock J.R. (2020). Novel Hybrid Molecules of 3,5-bis(benzylidene)-4-Piperidones and Dichloroacetic Acid Which Demonstrate Potent Tumour-Selective Cytotoxicity. Bioorganic Med. Chem. Lett..

[54] Saeed Abaee M., Mojtahedi M., Mehdi Zahedi M., Mohammad S. (2006). First Highly Efficient Synthesis of bis(Arylmethylidene)pyranones. Mediated by Lithium Perchlorate.Lithium Perchlorate. Synth. Commun..

[55] Saeed Abaee M., Mohammad M., Mehdi Zahedi M., Sharifi R.A. (2007). Highly Efficient Method for Solvent-Free Synthesis of Bisarylmethylidenes of Pyranonesand Thiopyranones. Heteroat. Chem..

[56] Madhusdana Reddy M.B., Nizam A., Pasha M.A. (2010). Molecular Iodine: An Efficient and Environment Friendly Catalyst for the Synthesis of 3,5-Bis-(arylmethylidene)- Tetrahydropyran-4-Ones. Synth. Commun..

[57] Nelson J., Debabrata C. (1957). 7-Pyrones by Isomerizaton. Substituted 3,5-Dibenzyl-4H-Pyran-4-Ones. J. Amer. Chem. Soc..

[58] Mardyukov A., Niedek D., Schreiner P.R. (2018). Unravelling Lawesson’s Reagent: The Structure of Monomeric (4-methoxyphenyl)phosphine Disulfide. Chem. Commun..

[59] Version 2016.10. https://www.molinspiration.com.

[60] Canavan N. (2007). FDA and Drug Companies Alike Want ADME-Tox Testing Performed Earlier and Earlier in a Drug’s Life Cycle. Drug Discov. Dev..

[61] ICM v3.8-5, 2016. https://www.molsoft.com.

[62] 2013. https://cyprules.cmdm.Tw.

[63] Version 2.0, 2008/10. https://preadmet.bmdrc.kr/.

[64] 2017. http://www.swissadme.ch.

[65] Veber D.F., Johnson S.R., Cheng H.-Y., Smith B.R., Ward K.W., Kopple K.D. (2002). Molecular Properties That Influence the Oral Bioavailability of Drug Candidates. J. Med. Chem..

[66] Kavetsou E., Katopodi A., Argyri L., Chainoglou E., Pontiki E., Hadjipavlou-Litina D., Chroni A., Detsi A. (2020). Novel 3-aryl-5-substituted-coumarin Analogues: Synthesis and Bioactivity Profile. Drug Dev. Res..

[67] Chu J., Praticò D. (2010). 5-Lipoxygenase As an Endogenous Modulator of Amyloid Beta Formation in Vivo. Ann. Neurol..

[68] Praticò D. (2008). Oxidative Stress Hypothesis in Alzheimer’s Disease: A Reappraisal. Trends Pharmacol. Sci..

[69] Chu J., Li J.-G., Ceballos-Diaz C., Golde T., Praticò D. (2013). The Influence of 5-Lipoxygenase on Alzheimer’s Disease-Related Tau Pathology: In Vivo and In Vitro Evidence. Biol. Psychiatry.

[70] Skrzypczak-Jankun E., Zhou K., Jankun J. (2003). Inhibition of Lipoxygenase by (-)-Epigallocatechin Gallate: X-Ray Analysis at 2.1 A Reveals Degradation of EGCG and Shows Soybean LOX-3 Complex With EGC Instead. Int. J. Mol. Med..

[71] Moody T.W., Leyton J., Martínez A., Hong S., Malkinson A., Mulshine J.L. (1998). Lipoxygenase Inhibitors Prevent Lung Carcinogenesis and Inhibit Non-Small Cell Lung Cancer Growth. Exp. Lung Res..

[72] Gleason M.M., Rojas C.J., Learn K.S., Perrone M.H., Bilder G.E. (1995). Characterization and Inhibition of 15-Lipoxygenase in Human Monocytes: Comparison With Soybean 15-Lipoxygenase. Am. J. Physiol. Physiol..

[73] Lapenna D., Ciofani G., Pierdomenico S.D., Giamberardino M.A., Cuccurullo F. (2003). Dihydrolipoic Acid Inhibits 15-Lipoxygenase-Dependent Lipid Peroxidation. Free. Radic. Biol. Med..

[74] Nuhn P., Büge A., Köhler T., Lettau H., Schneider R. (1991). Trends in the Development of Lipoxygenase inhibitors. Die Pharmazie.

[75] Maccarrone M., Van Aarle P.G., AVeldink G., Vliegenthart J.F. (1994). In Vitro Oxygenation of Soybean Biomembranes by Lipoxygenase-2. Biochim. Biophys. Acta (BBA)–Biomembr..

[76] Pontiki E., Hadjipavlou-Litina D. (2007). Lipoxygenase Inhibitors: A Comparative QSAR Study Review and Evaluation of New QSARs. Med. Res. Rev..

[77] Liargkova T., Hadjipavlou-Litina D., Koukoulitsa C., Voulgari E., Avgoustakis C. (2015). Simple Chalcones and Bis-Chalcones Ethers As Possible Pleiotropic Agents. J. Enzym. Inhib. Med. Chem..

[78] Mintzer J., Armstrong D., Faison W.E. (2003). Combination Pharmacotherapy in Alzheimer’s Disease. Neurocircuitry.

[79] Pontiki E., Hadjipavlou-Litina D. (2018). Multi-Target Cinnamic Acids for Oxidative Stress and Inflammation: Design, Synthesis, Biological Evaluation and Modeling Studies. Molecules.

[80] Sievers F., Wilm A., Dineen D., Gibson T.J., Karplus K., Li W., Thompson J.D., Higgins D.G., Lopez R., McWilliam H. (2011). A Scalable Generation of High-Quality Protein Multiple Sequence Alignments Using Clustal Omega. Mol. Syst. Biol..

[81] Liargkova T., Eleftheriadis N., Dekker F.J., Voulgari E., Avgoustakis K., Sagnou M., Mavroidi B., Pelecanou M., Hadjipavlou-Litina D. (2019). Small Multitarget Molecules Incorporating the Enone Moiety. Molecules.

[82] Pontiki E., Hadjipavlou-Litina D., Litinas K., Nicolotti O., Carotti A. (2011). Design, Synthesis and Pharmacobiological Evaluation of Novel Acrylic Acid Derivatives Acting As Lipoxygenase and Cyclooxygenase-1 Inhibitors With Antioxidant and Anti-Inflammatory Activities. Eur. J. Med. Chem..

[83] Fiser A., Šali A. (2003). Modeller: Generation and Refinement of Homology-Based Protein Structure Models. Methods Enzymol..

[84] Halgren T.A. (1996). Merck Molecular Force Field. I. Basis, Form, Scope, Parameterization, and Performance of MMFF94. J. Comp. Chem..

[85] Da Silva A.W.S., Vranken W.F. (2012). ACPYPE–AnteChamber PYthon Parser InterfacE. BMC Res. Notes.

[86] Wang J., Wang W., Kollman P.A., Case D.A. (2006). Automatic Atom Type and Bond Type Perception in Molecular Mechanical Calculations. J. Mol. Graph. Model..

[87] Lindorff-Larsen K., Piana S., Palmo K., Maragakis P., Klepeis J.L., Dror R.O., Shaw D.E. (2010). Improved Side-Chain Torsion Potentials for the Amber ff99SB Protein Force Field. Proteins Struct. Funct. Bioinform..

[88] Trott O., Olson A.J. (2009). AutoDock Vina: Improving the Speed and Accuracy of Docking With a New Scoring Function, Efficient Optimization, and Multithreading. J. Comput. Chem..

[89] UniProt (2019). A Worldwide Hub of Protein Knowledge. Nucleic Acids Res..

[90] Roy A., Kucukural A., Zhang Y. (2010). I-TASSER: A Unified Platform for Automated Protein Structure and Function Prediction. Nat. Protoc..

[91] Yang J., Yan R., Roy A., Xu D., Poisson J., Zhang Y. (2014). The I-TASSER Suite: Protein Structure and Function Prediction. Nat. Methods.

[92] Yang J., Zhang Y. (2015). I-TASSER Server: New Development for Protein Structure and Function Predictions. Nucleic Acids Res..

[93] Pettersen E.F., Goddard T.D., Huang C.C., Couch G.S., Greenblatt D.M., Meng E.C., Ferrin T.E. (2004). UCSF Chimera–a visualization system for exploratory research and analysis. J. Comput. Chem..

[94] http://ambermd.org.

[95] Maier J.A., Martinez C., Kasavajhala K., Wickstrom L., Hauser K.E., Simmerling C. (2015). ff14SB: Improving the Accuracy of Protein Side Chain and Backbone Parameters from ff99SB. J. Chem. Theory Comput..

[96] Jorgensen W.L., Chandrasekhar J., Madura J.D., Impey R.W., Klein M.L. (1983). Comparison of Simple Potential Functions for Simulating Liquid Water. J. Chem. Phys..

[97] Wang J., Wang W., Kollman P., Case D. (2001). Antechamber, An Accessory Software Package, For Molecular Mechanical Calculations. J. Am. Chem. Soc..

[98] Quiroga R., Villarreal M.A. (2016). Vinardo: A Scoring Function Based on Autodock Vina Improves Scoring, Docking, and Virtual Screening. PLoS ONE.

[99] Koes D.R., Baumgartner M.P., Camacho C.J. (2013). Lessons Learned in Empirical Scoring With Smina from the CSAR 2011 Benchmarking Exercise. J. Chem. Inf. Model..

