# Supplementary material for: Hybridization of Curcumin Analogues with Cinnamic Acid Derivatives as Multi-Target Agents Against Alzheimer’s Disease Targets
Source: Molecules. 2020 Oct 26;25(21):4958. doi: 10.3390/molecules25214958 (PMC7662280; doi:10.3390/molecules25214958)
Supplement: Supplementary file 1 [file molecules-25-04958-s001.pdf]

Table S-I. Molecular properties predictors calculated by the MolSoft platform [52, 91].

Comp.	TPSA (Å^2)[a]	Volume (Å^3)[b]	LogP	LogS (in mg/L)	Number of HBA	Number of HBD	%ABS =109-(0.345 x TPSA)[c]	Drug-Likeness Model Score (DLS)	BBB Score
**1a**	24.31	403.94	5.80 (> 5)	0.07	2	1	100.61	-1.36	4.64
**1b**	26.89	302.71	2.68	238.66	4	1	99.72	-0.72	5.54
**1c**	44.60	382.88	3.87	17.56	2	3	93.1	-1.64	4.11
**1d**	44.42	379.48	3.48	61.76	2	3	93.8	-1.42	4.07
**1e**	39.31	559.95	7.80 (> 5)	0.42	4	1	95.44	-1.22	3.93
**1f**	68.44	271.94	0.06	6010.37	4	3	85.39	-0.69	3.53
**1g**	24.86	306.30	3.48	53.22	2	1	100.42	-1.28	5.57
**1h**	26.89	336.64	3.94	19.86	4	1	99.72	-1.05	5.53
**1i**	26.89	350.95	4.07	23.47	4	1	99.72	-0.74	5.55
**1j**	24.86	318.13	3.61	36.11	2	1	100.42	-1.34	5.57
**2a**	28.62	151.60	2.22	522.96	2	1	99.13	-1.17	4.26
**2b**	28.35	200.43	3.19	68.87	2	1	99.22	-1.34	4.86
**2c**	61.72	174.88	1.27	3541.88	4	3	87.7	-0.35	2.80
**3a**	28.29	550.36	7.93 (> 5)	0.13	2	0	99.24	-0.75	3.43
**3b**	28.56	501.54	6.57 (> 5)	0.12	2	0	99.15	-0.75	3.81
**3c**	28.01	599.18	8.89 (> 5)	0.10	2	0	99.34	-0.79	3.27
**3d**	28.83	464.54	5.74 (> 5)	0.60	2	0	99.05	-0.56	4.40
**3e**	30.59	497.95	5.77 (> 5)	0.23	4	0	98.45	-0.55	3.87
**3f**	28.56	513.37	6.70 (> 5)	0.12	2	0	99.15	-0.35	3.72
**3g**	28.83	452.72	5.61 (> 5)	0.62	2	0	99.05	-0.76	4.49
**3h**	61.38	573.63	6.97 (> 5)	0.18	4	2	87.82	0.08	2.45
**1k**	12.86	394.39	6.86 (> 5)	0.03	1	0	104.56	-0.90	3.98
**1l**	21.94	299.34	3.91	14.25	2	0	101.43	-1.31	5.17
**1m**	21.40	396.98	6.23 (> 5)	0.05	2	0	101.62	-1.39	4.10
**1n**	12.49	413.39	7.22 (> 5)	0.04	2	0	104.69	-1.36	4.22

**1o**	11.71	313.93	4.18	11.10	2	1	104.96	-1.38	5.74
**1p**	11.17	411.57	6.50 (>5)	0.03	2	1	105.15	-1.47	4.81
**1q**	39.78	372.71	5.24 (> 5)	0.22	4	1	95.8	-1.23	4.12
**Curcumin (Keto)**	73.83	393.60	2.83	428.21	6	2	83.53	-0.82	2.83
**Curcumin (Enol)**	77.11	401.76	3.29	380.63	6	3	82.4	-0.61	2.78

[a]Topological polar surface area, [b]Molecular volume, [c]Percentage absorption

Number of HBA: Number of hydrogen bond acceptors (n-ON)

Number of HBD: Number of hydrogen bond donors (n-OHNH)

Data were calculated by the **MolSoftplatform** (http://molsoft.com/mprop/). The percentage of absorption (%ABS) was calculated by using %ABS =109-(0,345 x TPSA) and is referred to the degree of absorption.

All molecular property predictors are calculated using fragment-based contributions.
LogP (octanol/water partition coefficient)
LogS (water solubility)
Molecular Polar Surface Area (PSA) and Volume
PSA is defined as sum of surfaces of oxygens, nitrogens and attached hydrogens.
